# Associative memory of structured knowledge

**DOI:** 10.1038/s41598-022-25708-y

**Published:** 2022-12-17

**Authors:** Julia Steinberg, Haim Sompolinsky

**Affiliations:** 1 Department of Physics, Harvard University, Cambridge, MA 02138 USA; 2 Center for Brain Science, Harvard University, Cambridge, MA 02138 USA; 3 Joseph Henry Laboratories of Physics, Princeton University, Princeton, NJ 08544 USA; 4 Edmond and Lily Safra Center for Brain Sciences, Hebrew University, 91904 Jerusalem, Israel

**Keywords:** Long-term memory, Network models, Neural encoding

## Abstract

A long standing challenge in biological and artificial intelligence is to understand how new knowledge can be constructed from known building blocks in a way that is amenable for computation by neuronal circuits. Here we focus on the task of storage and recall of structured knowledge in long-term memory. Specifically, we ask how recurrent neuronal networks can store and retrieve *multiple* knowledge structures. We model *each* structure as a set of binary relations between events and attributes (attributes may represent e.g., temporal order, spatial location, role in semantic structure), and map each structure to a distributed neuronal activity pattern using a vector symbolic architecture scheme.We then use associative memory plasticity rules to store the binarized patterns as fixed points in a recurrent network. By a combination of signal-to-noise analysis and numerical simulations, we demonstrate that our model allows for efficient storage of these knowledge structures, such that the memorized structures as well as their individual building blocks (e.g., events and attributes) can be subsequently retrieved from partial retrieving cues. We show that long-term memory of structured knowledge relies on a new principle of computation beyond the memory basins. Finally, we show that our model can be extended to store sequences of memories as single attractors.

## Introduction

Human memory is remarkable in its ability to robustly store and retrieve information with complex and hierarchical structure, guiding cognitive processes on many different timescales. In many instances, this “structured knowledge” can be described as sets of associations between discrete events with their contextual attributes. Some concrete examples are, temporal sequences representing events associated to particular times, episodic memories representing events associated with particular contexts^1,2^, cognitive maps representing spatial environments through landmarks associated with locations^3–5^, and semantic structures in language in which meaning is conveyed through sets of words associated with their respective roles within a sentence^6–8^.

To effectively use structured knowledge that has been stored in long-term memory, it must be represented in a way that allows for its retrieval through partial information, with tolerance for noisy and degraded cues. This is likely facilitated by the distributed nature of the underlying neural representations, which provide an inherent notion of similarity between representations and a mechanism for learning representations by the the tuning of synaptic weights in a neural network^7,9–11^. However, while the utility of distributed representations is clearly beneficial from this perspective, it is still not well understood how to represent associations and relations in neural networks in an efficient and flexible way that is amenable to the variety of computational demands involved in higher cognition^12–14^.

Several recent studies have addressed the contextual modulation of neuronal representations, e.g., by forming “mixed representations”^15^ or by gating parts of the network^16^. Other proposals have tried to implement more general relational structures in neural networks. An early attempt used the tensor product to create a distributed representation of pairwise relations between discrete items^6^. Subsequently, several Vector-Symbolic Architectures (VSA) were proposed as compressions of the tensor product to avoid the increase in dimensionality of the representation, allowing for the creation of hierarchies of relations in a compact way^17–22^. More recently, several architectures for deep or recurrent neural networks have been proposed to promote flexible relational reasoning^23–30^. However, these works have primarily focused on working memory, i.e., online tasks of processing incoming structured data. By contrast, the challenge of storing and retrieving relational structures in long-term memory has received little attention.

Storing knowledge structures in long-term memory poses several additional challenges. While working memory tasks typically process few structures at a time, long-term memory networks must cope with storing a very large number of structures, such as complex cognitive maps, multiple sequences, or stories, which may scale with the size of the memory network itself. While several works in the psychology literature^31^ Two generic measures of the efficiency of information storage in recurrent neural networks are their extensive capacity, i.e., the number of stored items scales with the number of neurons in the network^32^, and the ability to recall memories from partial cues which have small but significant overlap with the desired memory^33^. Both of these measures can be adversely affected by correlations across memorized patterns. For relational structures, additional correlations may occur due to the presence of objects or contextual attributes in multiple memories, putting additional constraints on the encoding of relational information. In addition to these considerations, models of distributed representation of knowledge structures typically compress the relational structure into a fixed-length distributed vector representation. To compensate for this loss of information, “clean-up” mechanisms are invoked. Thus, it is crucial that such mechanisms can be adapted for the task of retrieval of such structures from long-term memory to efficiently store large numbers of relational structures each containing multiple associations.

In this work, we propose a model for associative memory of *multiple* relational structures by using a quadratic binding scheme to form vector representations of memories consisting of *multiple* binary relations between items (which we will henceforth denote as pairs of objects and their attributes).

While our model is quite general, in most of our work we will use the holographic reduced representation (HRR)^7^ VSA scheme for convenience. We show that the binarized versions of these structures can be stored as *fixed point* attractors in a recurrent neural network and each structure can be retrieved from the memory network by using a cue which is a structure encoding a subset of the relations in the memorized structure. We highlight the holistic nature of this model by comparing the storage of temporal sequences in the present model, where the entire sequence is stored as a single fixed point, to previous models, where a sequence is stored as a sequence of transitions between multiple fixed points and cannot be fully recalled at once^34^. Our model posits that in addition to the network that stores the structures, a Dictionary network stores all individual items (e.g., individual words, familiar objects). We show that the identities of the objects contained in the structure can be decoded faithfully from the retrieved memory by querying the retrieved structure with the appropriate cue as long as a “clean-up” operation is performed to map the noisy estimate of the object to the correct item in the Dictionary. Furthermore, this decoding works well even when the retrieved structure is significantly degraded.

## Relational structures

We begin by modeling a binary relational structure *S* as a set of *L* object/attributes pairs

1 $$\begin{aligned} S&=\lbrace (a_{1},b_{1}),\dots ,(a_{L},b_{L})\rbrace \end{aligned}$$

where both objects *a* and attributes *b* have embeddings as real vectors representing distributed patterns of activation in a neuronal population. For simplicity, both populations will be assumed to be of the same size *N*. We represent relations between items in a pair (*a*, *b*) by a transformation through a pairwise quadratic nonlinearity to a binding vector *g*(*a*, *b*) (in $\mathbb {{R}}^{N}$) representing a distributed pattern of activity in a population of *N* “binding” neurons. Each component of the binding vector takes the form

2 $$\begin{aligned} g_{k}(a,b)=a^TG^kb \end{aligned}$$

where each $G^{k}$ is an $N\times N$ fixed binding matrix. The binding operation in Eq. (2) is a generalized version of a VSA scheme^18^ and can be interpreted as a lossy compression of the tensor product binding operation first proposed in^6^.

We obtain the representation of the full relational structure *S* by the vector summation of the individual object/attribute pairs,

3 $$\begin{aligned} \widehat{S}&=\sum _{\ell =1}^{L}g(a_{\ell },b_{\ell }) \end{aligned}$$

where the vector summation induces a second source of information loss. The representation $\widehat{S}$ is permutation invariant with respect to the index $\ell$ so that the relations within the structure have no particular order.

The compressed representations of structures can be used for a variety of computations, such as structure classification. Here we focus on unbinding tasks, in which given $\widehat{{S}}$ and one of its attributes $b_{\ell }$, we need to estimate its pair $a_{\ell }$. Similar to binding, we assume that the unbinding operation is performed through a quadratic transformation involving the pair $(\widehat{{S}},b)$, so that the *k*-th component of the estimator $\hat{a}^k_{\ell }$ of $a_{\ell }$ is given by

4 $$\begin{aligned} \hat{a}^{k}_{\ell }&=\widehat{S}^TF^{k}b\,,\,k=1,...,N \end{aligned}$$

where each $F^{k}$ is an $N\times N$ matrix chosen so that the decoding operation is the approximate inverse of the binding operation.

In general, the binding and unbinding matrices can be learned and the optimal choice should depend on the nature of the items contained in the dictionary. Here we use a generic set of matrices, a popular choice known as Holographic Reduced Representations (HRR) described in "Methods".

The final estimate of *a*, $\tilde{a}$, is computed by comparing the noisy estimate against a Dictionary, i.e., a neural long-term memory system that stores all familiar objects $a_{d}$, using,

5 $$\begin{aligned} \tilde{a}&=\arg \max _{d\in \mathcal {D}}d\cdot \hat{a} \end{aligned}$$

**Figure 1:**
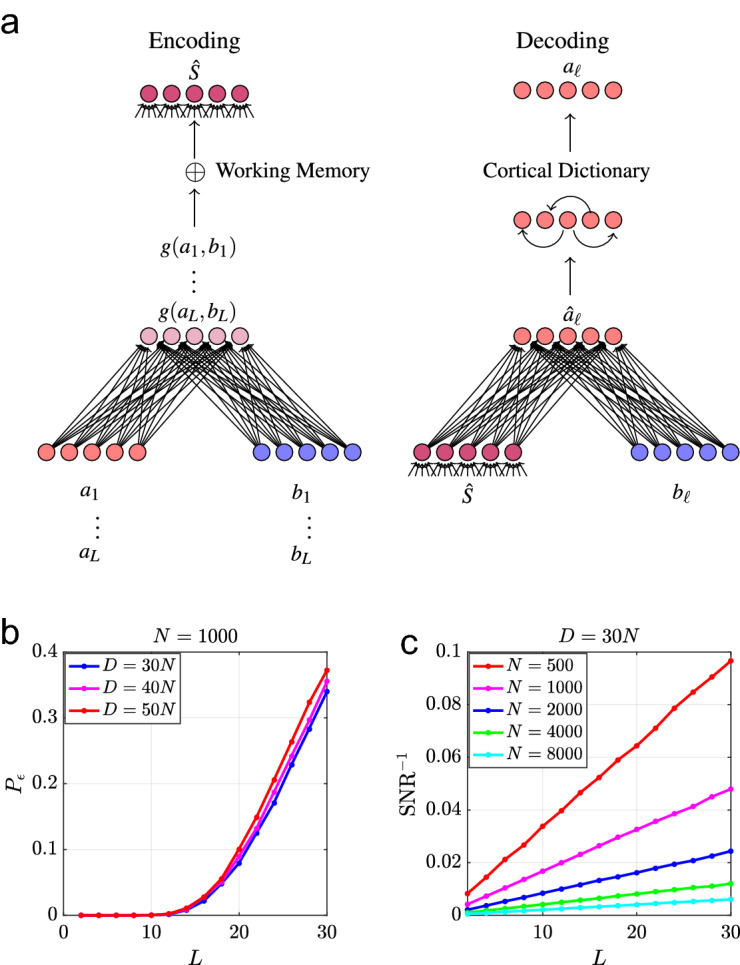
(**a**) A schematic of the network used to bind objects/attribute pairs (*a*, *b*) to form the knowledge structure $\widehat{S}$ alongside the network used to decode an object $a_{\ell }$ from $\widehat{S}^{\mu }$ by presenting the attribute $b_{\ell }$. (**b**) The unbinding error $P_{\epsilon }$ shown as a function of structure length *L* for $N=1000$ for various values of *D* averaged 10, 000 structures (**c**) $\text {SNR}^{-1}$ shown as a function of *L* for several values of *N* averaged over 10, 000 structures.

A schematic of the encoding and decoding networks is shown in Fig. 1a.

The maximum likelihood (ML) decoding error is given by the probability $P_{\epsilon }$ that the estimator $\hat{a}$ has the largest overlap with an incorrect item in the dictionary. It depends on the size of the dictionary *D* and the signal-to-noise ratio ($\text {SNR}$) overlaps of the estimator $\hat{a}$ with items dictionary defined in Eq. (20) in "Methods". ML decoding is an idealization of a more biologically realistic retrieval of the stored pattern *a* from a retrieval cue $\hat{a}$ in a long-term associative memory network storing all individual Dictionary items. Two possible implementations of this memory system are a winner take all network with lateral inhibition^35^ or a sparse Hopfield network^36^.

## Storing structures in long-term associative memory

**Figure 2:**
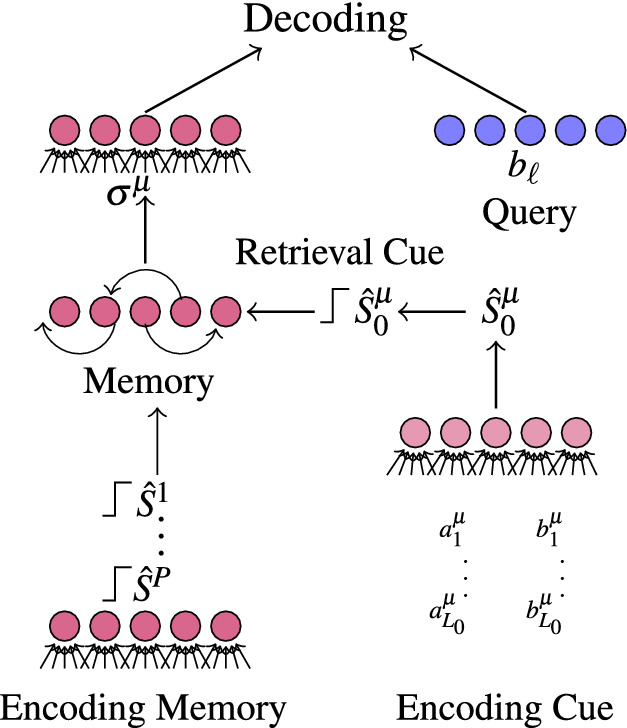
A schematic of the process of storing multiple binarized structures in a memory network. These structures can be retrieved from memory by encoding a retrieval cue from a subset of the relations in the desired structure as in Eq. (6) and initializing the network in the state of the binarized cue.

We now consider the long-term memorization of multiple knowledge structures by storing their vector representations in a neural network for long-term Structured Knowledge Associative Memory (SKAM), so that they can be retrieved at a later time from partial cues and subsequently queried to reconstruct individual events. We consider a set of *P* structures $\lbrace S^{1}, \dots , S^{P}\rbrace$ which for simplicity, all consist of *L* items. We label the set of L objects and attributes comprising the $\mu$-th structure as $a_{\ell }^{\mu }$, $b_{\ell }^{\mu }, l=1,...,L$.

For each structure, the HRR encoding scheme is used to create a vector representation $\widehat{S}^{\mu }$ from $S^{\mu }$.

To store multiple structures as fixed points in a neural network, the neuronal input-output transformation must be highly non-linear, implying that the stored patterns themselves are limited to the dynamic range of the neurons. As in a standard Hopfield network^32,33,37^, we assume neurons are binary $\pm 1$ variables and the memory patterns, candidates of fixed points of the attractor dynamics are $\sigma ^{\mu }={\text {sgn}}(\widehat{S}^{\mu })s$. For a network with *N* binary neurons, the memory load is defined as $\alpha =\frac{P}{N}$, where *P* is the number of stored structures.

In general, the associative nature of Hopfield memory networks is expressed in the ability to recall a memorized pattern starting in any initial state which has a sufficiently large overlap with the memorized pattern. If the initial state is within the basin of attraction of the pattern, it will converge to the pattern without errors. In our case, we consider partial cues of a structure, $\widehat{S}$, in the form of a recalling structure $\widehat{S}_{0}$ obtained by the binding and subsequent summation of any subset $S_{0}$ of the *L* pairs of binary relations contained in $\widehat{S}$, i.e.,

6 $$\begin{aligned} S_{0}^{\mu }&=\sum _{\ell =1}^{L_{0}}g(a_{\ell }^{\mu },b_{\ell }^{\mu }) \end{aligned}$$

where $L_{0}$ is the number of recalling elements in $\widehat{S}_{0}$ and is assumed to be much less than *L*. The network is then initialized in the state $\sigma _{0}^{\mu }=\text {sgn}(\widehat{S}_{0}^{\mu })$ and evolved to a fixed point which, if successful, corresponds to the stored binarized structure $\sigma ^{\mu }$. A schematic of our model is shown in Fig. 2 and more details on the memory network are given in "Methods".

There are several learning rules which can be used to store patterns as discrete fixed points in recurrent neural network models of associative memory. Here, for simplicity, we use the Pseudo-inverse learning rule proposed in^38^ to train the network. In a Pseudo-inverse network, all structures are perfect fixed points for $\alpha <1$, which is assumed throughout. This allows us to focus on the retrieval cues, since failure to perfectly recall a structure occurs only when the retrieval cue is outside of the basin of attraction of the memorized structure. We observe qualitatively similar behavior for the Hebb learning rule and the Storkey rule introduced in^39^ for $\alpha$ well below the memory capacity described in Section 3.4 of the Supplementary Material.

## Results

We evaluate the performance of the scheme introduced in the previous section by the ability to accurately perform the unbinding operation after retrieval of a structure from the SKAM. For example, after retrieving the structure $\mu =1$, we should be able to extract the item $a_{\ell }^1$ with a query in the form of its pair, i.e., $b_{\ell }^1$ with low error. We quantify performance by the average unbinding error $P_{\epsilon }$ obtained in simulations where structured memories are created from random patterns, stored in memory, retrieved with partial cues $\sigma _{0}^{\mu }=\text {sgn}(\widehat{S}_{0}^{\mu })$, and subsequently decoded using the ML “clean-up” operation. We assume all items appearing in memorized structures are stored in dictionaries for objects and for attributes which are then used to decode from the retrieved memory. A schematic of this process is shown in Fig. 2 and full details of the simulations are provided in "Methods".

The parameters involved in the performance measure are: network size *N*, memory load $\alpha =P/N$, size of the relational structures and the retrieval cue, denoted as *L* and $L_{0}$, respectively. We consider the regime where both *N* and *P* are very large and the memory load $\alpha \sim O(1)$^32,40^, mainly considering values $\alpha \sim 0.1-0.2$, where the network acts as a good associative memory.

### Retrieval of structured memories

**Figure 3:**
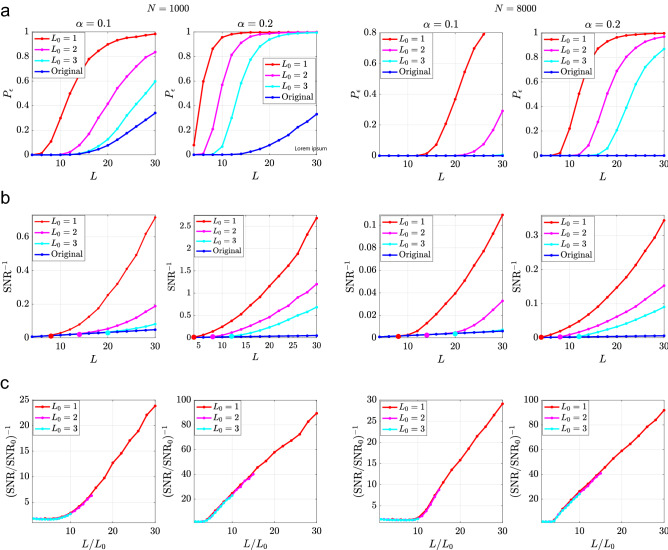
(**a**) The decoding error $P_{\epsilon }$ is compared for several values of $L_{0}$ from structures containing *L* pairs of items for two values of *N* and two values of $\alpha$. (**b**) $\text {SNR}^{-1}$ v. *L*. For each value of $L_{0}$, $l_{c}$ is given by the value of *L* where $\text {SNR}^{-1}$ diverges from the line corresponding to the original structure, which is marked for each value of $L_{0}$. (**c**) $\left( \text {SNR}/\text {SNR}_{0}\right) ^{-1}$ v. $\frac{L}{L_{0}}$ where $\text {SNR}_{0}=\frac{N}{L}$. For all figures, $T=20$ parallel updates are used in memory retrieval and the average is performed over 10, 000 memories. The dictionary is fixed to $D=30N$.

We begin by showing numerical results which measure the quality of the retrieved structures in terms of the unbinding error $P_{\epsilon }$ and the SNR of overlaps defined in Eq. (20). In all reported results, the extracted item (and the associated query) comes from pairs that are not part of the cueing structure $S_{0}$. Thus any performance better than chance necessarily involves information extracted by the retrieval from long-term memory. In Fig. 3a we show the dependence of the unbinding error $P_{\epsilon }$ on *L*, $L_{0}$, and $\alpha$. For comparison, we show $P_{\epsilon }$ for the original structure prior to storage in the memory network, demonstrating that except for small *L*, the dominant contribution to the error comes from retrieving the structure from long-term memory. We also observe that for a fixed *L*, $L_{0}$, and $\alpha$, the error is suppressed as *N* increases, in contrast to standard large attractor memory networks where performance depends only on *P*/*N*. To elucidate this behavior, we replot the results in terms of the $\text {SNR}^{-1}$ , i.e., the inverse of the SNR as defined above in Fig. 3b, showing that for each $L_{0}$, there is a critical *L* above which the SNR of the memorized structures decreases relative to the original SNR, $\text {SNR}_0$ before storage in long-term memory. Note that due to binarization, $\text {SNR}_0$ is smaller by a factor of $\frac{2}{\pi }$ relative to the value given in Eq. (22). we replot the same results in terms of the inverse of the normalized SNR, $\text {SNR}/\text {SNR}_{0}$ vs., $L/L_{0}$. Since $\text {SNR}_{0}$ is proportional to *N*/*L*, this normalization factors out the “trivial” dependence on *L*/*N* from the post retrieval $\text {SNR}$. Figure 3c shows that for a fixed $\alpha$ the normalized inverse SNR depends only on $L/L_{0}$. and only weakly on *N*, suggesting that the main *N* dependence comes from the linearity of $\text {SNR}_{0}$ in *N*.

### Length of cueing structure and memory basins

As seen in Fig. 3, the performance worsens (and SNR decreases) as *L* increases, while the converse holds true for $L_{0}$. We find that there is a critical ratio, $l_{c}=\frac{L_{0}}{L}$, defined as the minimum initial cue (relative to the total length), that leads to very small error which is essentially equivalent to the error for the original structure.

To understand the origin of $l_{c}$, we note that $\frac{L}{L_{0}}$ specifies the average initial overlap of the retrieval cue with the corresponding memorized structure, which we denote $m_{0}$. For small values of $L_{0}$, $m_{0}\approx \frac{2}{\pi } \sqrt{\frac{L_{0}}{L}}$. The size of $m_{0}$ determines whether on average the initial state is within the basin of the desired memory, so that the recurrent dynamics will succeed (or fail) in converging to the desired attractor. As the cue length $L_{0}$ grows, the initial state becomes increasingly likely to be within the basin of attraction of the desired structure, retrieving it with essentially no error. In these conditions, the unbinding operation has the same probability of success as for the original structure. Conversely, for small enough $L_0$ the initial state is likely outside the attraction basin of the memory, leading to errors in the retrieved structure.

To determine the minimum value of $L_0$ required for perfect retrieval, we use known estimates^38^ of the radius of attraction in attractor memory networks, $R(\alpha )=1-m_{min}(\alpha )$, where $m_{min}(\alpha )$ is the minimal overlap between the initial state and the desired memory required for convergence to the correct fixed point on average. $m_{min}(\alpha )$ determines the minimal length of the cueing structure, i.e., $l_c(\alpha )\approx \frac{\pi }{2} m_{min}^2(\alpha )$.

We conclude that when $L_{0}/L<l_{c}$, the main source of the decoding error in our model comes from the limitation on good retrieval of the structure from memory, due to small values of $L_0/L$, and not from noise in the original encoding (corresponding to $L_0/L>l_c$).

### Retrieval outside memory basins

Naively, one would expect that for $L_{0}<l_{c}L$, $P_{\epsilon }$ will be very large due to the accumulation of errors in the retrieved structure, which is outside the memory basin. However, as shown in Fig. 3, this is not case. Surprisingly, the decoding performance is well below chance level for substantial range of values of *L*, even when $L\gg L_{0}/l_{c}$. This observation can be explained by two scenarios: (1) the actual basins fluctuate in their shape so that for some structured memories, initial states may converge to the memory fixed point even if they are outside the *mean* basin radius; (2) initial states outside the true memory basin converge to fixed points with significant overlap with the structured memory.

To test these scenarios, we measured the empirical distributions *p*(*m*) where *m* is the overlap between the fixed point and the desired structure, obtained from histograms of overlaps for several values of $L>\frac{L_{0}}{l_{c}}$, shown in Supplementary Fig. 3.

**Figure 4:**
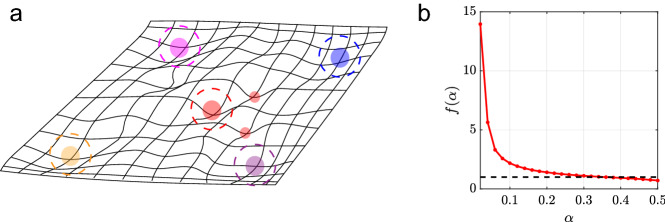
(**a**) schematic of the energy landscape of the memory network. The large filled circles are stored memories and the dashed circles denote their basins of attraction. The two small red circles are fixed points outside of the basin of attraction of the red memory which still lead to a large enough overlap for accurate decoding when $1\gg N/L$. (**b**) $f(\alpha )$ defined in Eq. (7) is obtained from simulations of a Pseudo-inverse network storing random memories of size $N=8000$. The black dotted line at 1 shows that $m^{*}>m_{0}$ for $\alpha \lesssim 0.3$. $T=20$ parallel updates are used for memory retrieval and averages are performed over 50 trials.

We find that as *N* is increased, *p*(*m*) becomes sharply peaked around a single value $m^{*}$. Inside the basin of attraction, i.e., $m_{0}<m_{min}(\alpha )$, $m^{*}=1$. However, outside of the basin when $m_{0}<m_{min}(\alpha )$, $m^{*}<1$; nevertheless it is substantially larger than 0. The value of $m^{*}$ depends on both $m_0$ and the load $\alpha$ roughly as

7 $$\begin{aligned} m^{*}(\alpha , m_0)&\sim f(\alpha )m_{0} \end{aligned}$$

described in further detail in "Methods" and the Supplementary Material 3. A schematic of the energy landscape is shown in Fig. 4a. Furthermore, for $\alpha \lesssim 0.3$, $f(\alpha )>1$ (Fig. 4b), implying that the final overlap with the retrieved structure is significantly larger than the initial overlap $m_{0}$ even far outside the basin of the structure.

### SNR of retrieved structures outside the basin

**Figure 5:**
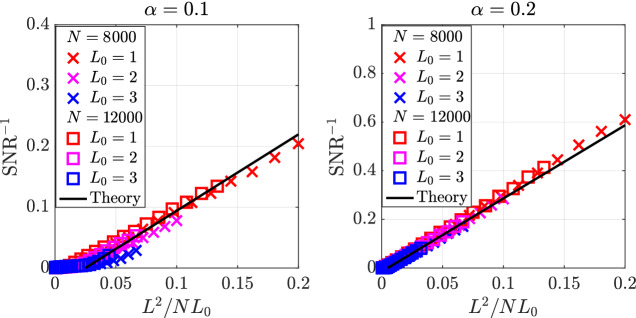
$\text {SNR}^{-1}$v. $L^{2}/NL_{0}$ shown for $N=8000$ and $N=12000$ and for several values $L_{0}$ as an average over items contained in 10, 000 memories shown for $\alpha =0.1$ and $\alpha =0.2$. The black line is obtained from Eq. (9) with $c=0.65$. $T=20$ parallel updates are used for memory.

We use the preceding results to estimate the SNR for $L_{0}/L\ll l_{c}$, i.e., when the initial state is well outside the memory basin. First, we argue that the SNR of unbinding from a noisy state with overlap $m<1$ with the true structure, should be roughly,

8 $$\begin{aligned} \text {SNR}(m)&\sim \frac{2c}{\pi }\frac{m^{2}N}{L} \end{aligned}$$

where, as before, the factor of $\frac{2}{\pi }$ comes from binarization and $c\approx 0.65$ accounts for the fact that part of the overlap *m* is contributed by the initial cueing structure $S_0$ and is more concentrated around the relations contained in the retrieval cue. For very large networks, we can replace *m* in Eq. (8) with $m^{*}$ from Eq. (7). Using Eq. (30) from "Methods", we express $m_{0}$ in terms of $L_{0}/L$ and arrive at

9 $$\begin{aligned} \text {SNR} \sim \frac{8cf(\alpha )^{2}}{\pi ^{3}}\frac{N L_0}{L^2},\,L_{0}\ll L \end{aligned}$$

which is verified in Fig. 5 for two values of $\alpha$. These results summarize the rich behavior of associative memory of structured knowledge. In contrast to standard memory functions, here the performance depends not only on the memory $\alpha$ but also on the network size *N*, structure length *L*, and cueing length $L_0$ through the SNR. The key difference is that in structured memories, the criterion for success is not limited to convergence to the target memory; even if the target memory is only partially retrieved, the underlying memorized relations can be still be retrieved faithfully using the semantic memory. The well-known property of pattern completion is realized here by a sub-structure of length $L<L_0$, in addition to the standard random initial condition.

## Storage and retrieval of sequences

### Storing sequences as binary structures

We now extend the results of the previous sections to representations of temporal sequences. Temporal sequences can be modeled as structures in several ways. One possibility is to bind each event in the sequence with its temporal order in the sequence. This can be implemented via a contextual drift process with a context representation that evolves as items in the sequence are retrieved as in the Temporal Context Model of free recall of lists^2^ and similarly in the Context Retrieval and Updating model^41,42^. Here we use temporal proximity as the contextual cue by interpreting a sequence as a set of binary associations between temporally proximal events. Thus, a temporal sequence of length *L*, $(a_{1},a_{2},...,a_{L})$ can be represented as a structure of the form

10 $$\begin{aligned} S&=\lbrace (a_{1},a_{2}),(a_{2},a_{3}),\dots ,(a_{L-1},a_{L})\rbrace \end{aligned}$$

and the entire sequence *S* is represented by a vector $\widehat{S}$ of size *N* given by

11 $$\begin{aligned} \widehat{S}&=\sum _{\ell =1}^{L-1}g(a_{\ell },a_{\ell +1}) \end{aligned}$$

Decoding an episode at a particular time, i.e., $a_{\ell }$, is performed through an unbinding operation with a query by the preceding event, $a_{\ell -1}$. Starting from a query by $a_{1}$, the entire sequence can be unfolded through a sequence of queries. Because each event appears in two binary relations, we need to use an asymmetric binding operation so that $g(a,b)\ne g(b,a)$. Within HRR, this can be accomplished by switching the binding and unbinding operations^43^.

**Figure 6:**
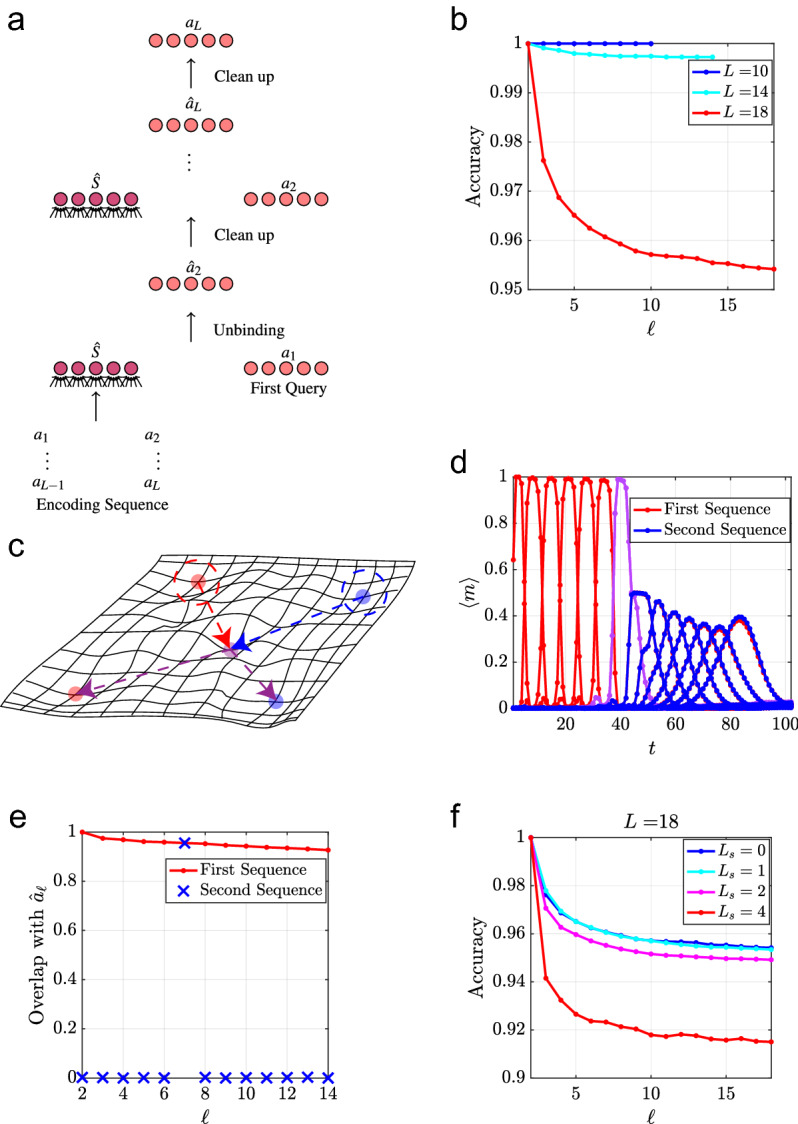
(**a**) A schematic of the decoding scheme for sequences. (**b**) Decoding accuracy as a function of sequence position $\ell$ along sequences of length *L* retrieved from memory shown for several values of *L*. $N=2500$, $\alpha =0.05$, and the average is computed over 100 trials. (**c**) A schematic of the energy landscape for two temporal sequences containing a single common element which are encoded as sequences of attractors using the scheme in^34^ (discussed in Section 4 of the Supplementary Material). (**d**) The average overlap of the network state $\langle m \rangle$ with each attractor in the sequence is shown as a function of retrieval time for a network of size $N=1000$ storing two sequences containing a single element in common. The parameters $\tau =8$ and $\lambda =2.5$ are used and the average is computed over 1000 trials. (**e**) The average overlap of the estimator $\hat{a}_{\ell }$ (normalized by $\langle \hat{a}_{2}\cdot a_{2}\rangle$) with the correct Dictionary item at each position in the sequence for a network of size $N=1000$ storing two sequences containing a single element in common. The average is computed over 10, 000 trials. (**f**) Decoding accuracy as a function of sequence position $\ell$ for $P=\alpha N$ sequences for which each neighboring sequence contain $L_{s}$ common elements with the previous one. $N=2500$, $\alpha =0.05$ and the average is computed over 100 trials.

As before, we consider the case in which all items being decoded are contained in a Dictionary $\mathcal {D}=\left\{ a_{1},a_{2},\dots ,a_{D}\right\}$, so each decoding step involves a clean-up of the decoded item before preceding to decode the next item from the sequence. A schematic of this process is shown in Fig. 6a.

The binarized versions of the structures representing each sequence are stored for long-term memory in a recurrent neural network with synaptic weight matrix determined via the Pseudo-inverse rule. The cueing structure $\widehat{S}_{0}$ consists of the first relation $(a_{1},a_{2})$, so the overlap of $\widehat{S}_{0}$ with the stored sequence $\widehat{S}$ is closely approximated by Eq. (30) with $L_{0}=2$. Alternatively, the first item $a_{1}$ can be used as a retrieval cue if it is added to the representation $\widehat{S}$ in Eq. (11). We are primarily interested in the ability to reconstruct the entire sequence after it is retrieved from memory.

### Retrieval of sequences from long-term memory

Due to the sequential nature of decoding sequences in our model, the decoding error accumulates as each subsequent element is retrieved. Thus, the unbinding error for an event depends on its position in the sequence (relative to the cued events). In Fig. 6b, we show the decoding error $P_{\epsilon }$ at each position along sequences encoded by Eq. (11) for sequences of different length *L*. Since the SNR of the overlap with the correct item along each position in the sequence depends on *L*, the length of the sequence limits the accuracy of decoding at all positions along the sequence. Nevertheless, for low memory loads and moderately long sequences, the accumulated error is small.

### Decoding error

An interesting outcome of this mode of recall is the accumulation of errors as the recall sequence advances. This would give rise to correlations between probabilities of recall that decay as a function of the temporal lag between the events, consistent with observations^2^. This behavior was previously explained by positing that proximal temporal context vectors are correlated. In our model, these correlations are a natural consequence of the fact that the proximal events serve as temporal context cues.

The present scheme of long-term storage of sequences as single fixed points overcomes a key disadvantage of previous attempts at storing multiple temporal associations in attractor neural networks. In previous models of sequential memory^34^, all of the patterns contained in the sequences are stored as separate attractors in one network and the sequences themselves are encoded in a time-delayed synaptic plasticity rule that associates each pattern with its next pattern in the sequence, illustrated in Fig. 6c and reviewed in Section 4 of the Supplementary Material. Because of the Markovian nature of the synaptic plasticity, retrieval will fail if multiple sequences share the same event, as shown in Fig. 6d. By contrast, in the present model, the entire sequences are stored and retrieved as separate “holistic” attractors. Thus, as long as the retrieval cue is unique to a single sequence, it will retrieve it unambiguously and the subsequent unfolding of the sequence by the unbinding network will be immune from interference with other sequences. To demonstrate this, we consider *P* sequences $S^{\mu }$ with $\mu =1,\dots ,P$ of length *L* where neighboring sequences $S^{\mu }$ and $S^{\mu +1}$ share $L_{s}$ events in common.

In Fig. 6e, we show the decoding error for a sequence stored in memory with another sequence containing an overlapping event, demonstrating the successful retrieval of the entire sequence despite the presence of an overlapping state (compare with Fig. 6d).

Figure 6f shows that sequences can be faithfully retrieved even with multiple common states, up to the point where the basins of attraction of individual sequences shrink due to the large overlap between them.

Finally, it is interesting to compare the the memory capacity of the two models. In the sequence attractor model, the maximal number of stored sequences of length *L* is $P<\alpha _c N/L$ since the network stores *PL* states. In contrast, in the present model, since only *P* attractors are stored, the capacity of storage is $P<\alpha _c N$. Nevertheless, for a successful unfolding of the sequence we need *PL*/*N* to be bounded (for a fixed $L_0/L$) due to the noise in the unbinding operation. A potential disadvantage of the current model is the need to devote additional memory resources to store the individual events in a Dictionary. On the other hand, the Dictionary can be used for multiple other cognitive tasks aside from the retrieval of these sequences. Another flexibility in the separation of the retrieval of the neural representation of the sequence from the subsequent reconstruction of individual events, is the fact that, for some tasks, the agent may not need to access the full detail of the sequence, for instance in tasks that requires distinguishing between one episode and another one. Such tasks may not need to rely on the full unbinding of the sequences.

## Neural implementation of multiplicative binding

We now briefly consider possible implementations of the binding computation in Eq. (2) through multiplicative interactions in biological neurons^44^. Previously, several mechanisms have been proposed to facilitate multiplicative interactions among neurons including dendritic gating^45^, quadratic firing rate response^46^, and short-term synaptic plasticity. Short-term plasticity comprises a variety of synaptic processes that modulate synaptic efficacy in a transient, activity-dependent manner^47^. These processes occur on timescales ranging from milliseconds to minutes^48^ and are thought to mediate higher cognitive functions like attention and working memory. More recently it has been suggested that “fast weights” in artificial neural networks may serve as an analogy to short-term plasticity in the brain^49^ with connections to linear transformers^50^.

We start by noting that $g_{k}(a,b)=a^Tb'$ where $b'=G^kb$. The last term is a representation of the activity pattern *b* by propagating it through a synaptic matrix $G^k$. Finally the dot product between *a* and $b'$ can be implemented can be decomposed into an outer product of two fixed synaptic weight vectors, i.e. $G^{k}=w^{k}_{a}w^{kT}_{b}$ so that components of the binding vector take the form

12 $$\begin{aligned} g_{k}(a,b)=(w^{k}_{a}\cdot a)(w^{k}_{b}\cdot b) \end{aligned}$$

We now use the above form to consider how firing rate nonlinearity and short-term synaptic plasticity can serve as mechanism for generating quadratic binding.

### Nonlinearity of the firing rate

Biological neurons can potentially implement the computation of the binding vector *g*(*a*, *b*) via the nonlinearity of the firing rate response to a synaptic current $r=f(I)$ where the synaptic current is given by the sum $I=w^{k}_{a}\cdot a+w^{k}_{b}\cdot b$. While for many neurons rectificed nonlinearity $f(I)=[I-I_{0}]_{+}$ is a good approximations, other neurons are found to be approximated by quadratic nonlinearity $f(I)=[I-I_{0}]^{2}_{+}$ where the firing in response to the sum of the separate responses to each input *a* and *b* is subtracted from the response to the combined input from *a* and *b*. Building on a quadratic *f*(*I*) curve, we can write

13 $$\begin{aligned} f(w^{k}_{a}\cdot a+w^{k}_{b}\cdot b)-f(w^{k}_{a}\cdot a)-f(w^{k}_{b}\cdot b)=2(w^{k}_{a}\cdot a)(w^{k}_{b}\cdot b) \end{aligned}$$

The subtraction can be implemented by inhibitory neurons or by temporal derivative in a working memory system.

### Short-term synaptic plasticity

Another potential mechanism to generate quadratic binding is short-term synaptic plasticity. This can be accomplished by a short-term increase in residual presynaptic calcium levels in working memory enabling *b* to modulate the synapses $G^{k}$ so that subsequent input $a^{T}$ will generate the postsynaptic potential

14 $$\begin{aligned} \omega (w^{k}_{a}\cdot a)+(\omega +\Delta \omega )(w^{k}_{b}\cdot b)&\approx \omega (w^{k}_{a}\cdot a+w^{k}_{b}\cdot b) \nonumber \\&+(w^{k}_{a}\cdot a)(w^{k}_{b}\cdot b) \end{aligned}$$

which contains a multiplicative component of the form in Eq. (12). Note that Eq. (14) contains a linear term weighted by $\omega$. This term may not need to completely cancel as it provides the trace with some similarity to both *a* and *b*, potentially allowing objects or context to be independently used as a retrieval cue. However, if $\omega$ relatively small, the trace will remain most similar to the bound conjunction *g*(*a*, *b*).

## Discussion

In summary, we have proposed and analyzed a model demonstrating how multiple knowledge structures, i.e., sets of relations between pairs of items, can be represented, stored, and retrieved in Hopfield type recurrent neural network models of long-term memory. Our model hypothesizes that the entire set of relations is encoded through binding operations, summation and binarization, in a single pattern of activity in a neuronal population, which is then stored as fixed point in the recurrent network. Retrieval of relational information from long-term memory, consists in our model of two stages: first, retrieval of the desired fixed point, and subsequent unbinding to uncover individual relations with the aid of a separate memory system, the Dictionary. Our analysis of this model clearly shows that the decoding $\text {SNR}$ exhibits the appropriate scaling of parameters required for accurate decoding of objects coexisting among many other relations within a structure, and also among the extensive number of other structures stored in memory.

We also show that this scheme can be used to model long-term memory of temporal sequences by creating structure vectors for sequences of temporally associated items and store in a recurrent network compressed versions of the sequences as fixed points. Sequence recall consists of retrieval of the “sequence” fixed point, and subsequent unfolding of the stored events through a sequence of unbinding operations. In this application we have also demonstrated that our model for storing structure vectors in long-term memory is not very sensitive to the presence of a partial overlap between different structures.

Our analysis suggests that the success of this long-term memory system depends not only on the memory capacity of the attractor network but also very crucially on the properties of the memory basins of attraction and the landscape in the surrounding “terrain”, such as the degree of overlap between “spurious” states outside the basins with the target fixed point (inside it). For this reason, a learning rule that decorrelates memories and yields smoother basins is clearly superior, as shown in Supplementary Fig. 8. Due to the dense distributed nature of the binding scheme employed here (HRR), we have not studied the effect of pattern sparsity on the long-term memory system^36^. It would be interesting to explore the sparsity effect in sparse binding schemes^51–54^ and generally how the binding matrices can be learned in a biologically plausible way.

We close by briefly discussing two important aspects of this work which have the most immediate phenomenological implications. A key aspect of our model is the existence of neuronal populations representing entire relational structures in long-term memory as persistent patterns of activity displaying the “holistic context” of each structure. This system interacts with a working memory system which executes the dynamics of retrieving details of the stored relations. We have not addressed the interesting question of the mechanism by which a stream of experiences is segmented into a sequence of discrete events^55^, or more generally, the mechanism that segments complex environments into a discrete sets of bound items and how these representations may evolve over time^56,57^. In particular, our model of long-term memory of sequences predicts that the retrieval of a temporal sequence is associated with a persistent pattern of activity (representing the context of the entire sequence) in addition to sequential dynamics involving the dynamic interaction between working and long-term memory. This can be tested in recordings of neuronal activity during recall of sequences in the hippocampus and in songbirds. It would also be interesting to see how this fits in with studies of the dynamics of recognition memory in the psychology literature^56,58^.

Finally, as mentioned above, our framework of storage and retrieval of relational knowledge structures in long-term memory relies on the existence of a complementary long-term memory system, the “Dictionary”, which stores the individual building blocks comprising the relational knowledge. It is tempting to identify these two complementary memory systems as representing episodic memory (the relational system)^1^ and semantic memory (the Dictionary)^59^, although we emphasize that in the present context, semantic memory does not necessarily require language and presumably exists in other species as well. The synergy of these two “complementary” memory systems results in an associative memory system with both the capacity and flexibility to store and faithfully represent complex knowledge structures in long-term memory in analogy with the “complementary learning systems” framework proposed in^60^ and revisited in^61,62^. Adapting this framework to further explain empirically observed phenomena in memory will require adherence to known biological properties of hippocampal representations as well more explicit models of both the Dictionary and the working memory system in which binding and unbinding occurs.

## Methods

### Holographic reduced representation

HRR^8,63^ is a commonly used VSA scheme with fixed forms for the binding and unbinding matrices in Eqs. (2) and  (4). The binding operation *g* is given by the circular convolution operation of the vectors *a* and *b* where

15 $$\begin{aligned} g_{k} =\sum _{j=0}^{N-1}a_{j}b_{k-j}\;,\,k=0,\dots ,N-1 \end{aligned}$$

and all subscripts are defined modulo *N*. The circular convolution operation is both associative and commutative. The corresponding decoding operation $\phi$ is realized through circular correlation of the two vectors $\widehat{S}$ and *b* where

16 $$\begin{aligned} \hat{a}_{k\ell } =\sum _{j=0}^{N-1}\widehat{S}_{j}b_{j+k,\ell } \end{aligned}$$

We see from comparing Eqs. (2) and (4) with Eqs. (15) and (16) that HRR corresponds to the following choice for the encoding and decoding matrices

17 $$\begin{aligned} G_{ij}^{k}&=\delta _{k,j+i}\end{aligned}$$

18 $$\begin{aligned} F_{ij}^{k}&=\delta _{k,j-i} \end{aligned}$$

The commutativity of the encoding operation implies that HRR encoding is commutative. To represent non-commutative asymmetric relations, we can simply exchange the binding and unbinding operations i.e., binding with circular correlation and unbinding with circular convolution^43^. The full details of the statistics of decoding for HRR is given in Section 1.2 of the Supplementary Material.

### Unbinding accuracy

We access the typical decoding performance by considering the case in which *a*’s and *b*’s are random vectors with components drawn iid from $\mathcal {N}(0,\frac{1}{N})$ and the dictionaries for *a*’s contains *D* elements. Then the ML decoding error is well approximated by

19 $$\begin{aligned} P_{\epsilon }&\approx \int _{-\infty }^{\infty }Dz\left( 1-H\left( -z-\sqrt{\text {SNR}}\right) ^{D}\right) \end{aligned}$$

where $Dz=\frac{dz}{\sqrt{2\pi }}e^{-\frac{z^{2}}{2}}$ and $H(z)=\frac{1}{2}\text {erfc}\left( \frac{z}{\sqrt{2}}\right)$. Here SNR is a signal-to-noise-ratio defined in terms of the mean overlap of the estimator $\hat{a}_{d}$ with the correct Dictionary item $a_{d}$ and the variance of the overlap with incorrect Dictionary item $a_{d^{\prime }}$, i.e.,

20 $$\begin{aligned} \text {SNR}&=\frac{\langle \hat{a}_{d}\cdot a_{d}\rangle ^{2}}{\langle (\hat{a}_{d}\cdot a_{d^{\prime }})^{2}\rangle } \end{aligned}$$

where $d^{\prime }\ne d$, and the average is over the Gaussian distributions of the components of $a_{d}$ and $a_{d^{\prime }}$. For full details see Section 2 of the Supplementary Material. For $\text {SNR}\gg 1$, the decoding error can be approximated as

21 $$\begin{aligned} P_{\epsilon }&\approx \sqrt{\frac{2}{\pi \text {SNR}}}De^{-\frac{\text {SNR}}{4}} \end{aligned}$$

To leading order, the SNR for many VSA binding schemes (including HRR) is

22 $$\begin{aligned} \text {SNR}&\sim \frac{N}{L} \end{aligned}$$

Equation 21 implies that $P_{\epsilon }\ll 1$ as long as $N\gtrsim O(L\log D)$. Hence, for $L\ll N$ accurate decoding requires the size of the Dictionary *D* be at most polynomial in *N*. In this regime, assumed throughout, the size of the Dictionary has little effect on performance, which is dominated by the SNR. $P_{\epsilon }$ and the inverse SNR are shown as functions of *L* in Fig. 1b and c, respectively.

### Memory network

Throughout this work, we consider Hopfield type recurrent neural networks with binary neurons. The state of the network at time *t*, $\sigma (t)$ is given by the update rule

23 $$\begin{aligned} \sigma _{i}(t)&=\text {sgn}\left( \sum _{j}J_{ij}\sigma _{j}(t-1)\right) \end{aligned}$$

where updates are done either in series or in parallel. For simplicity, parallel updates are used for the figures in the main text, but we show in Section 3.3 of the Supplementary Material that the results are qualitatively similar for serial updates.

Given a set of memories $\sigma ^{\mu }$, the synaptic weight matrix $J_{ij}$ must be chosen so that each of the memories is a fixed point of the dynamics in Eq. (23). There are several different learning rules which can accomplish this. Mainly, we consider the Pseudo-inverse rule^38^ with synaptic weight matrix given by

24 $$\begin{aligned} J_{ij}&=\frac{1}{N}\sum _{\mu ,\nu =1}^{P}\sigma _{i}^{\mu }C_{\mu \nu }^{-1}\sigma _{j}^{\nu },\,\,J_{ii}=0 \end{aligned}$$

where the pattern overlap matrix $C_{\mu \nu }$ is defined as

25 $$\begin{aligned} C_{\mu \nu }&=\frac{1}{N}\sum _{i=1}^{N}\sigma _{i}^{\mu }\sigma _{i}^{\nu } \end{aligned}$$

We also consider the Hebb rule given by

26 $$\begin{aligned} J_{ij}&=\frac{1}{N}\sum _{\mu =1}^{P}\sigma _{i}^{\mu }\sigma _{j}^{\mu },\,\, J_{ii}=0 \end{aligned}$$

and the Storkey rule^39,64,65^ with $J_{ij}$ given by

27 $$\begin{aligned} J_{ij}^{\mu }&=\frac{N+1}{N-1}J_{ij}^{\mu -1}+\frac{1}{N-1}(\sigma _{i}^{\mu }\sigma _{j}^{\mu }-\delta _{ij}) \nonumber \\ {}&-\frac{1}{N-1}(\sigma _{i}^{\mu }h_{j}^{\mu }+\sigma _{j}^{\mu }h_{i}^{\mu })\nonumber \\ J^{\mu }_{ii}&=0 \end{aligned}$$

where,

28 $$\begin{aligned} h_{i}^{\mu }&=\sum _{k=1}^{N}J_{ik}^{\mu -1}\sigma _{k}^{\mu } \end{aligned}$$

These learning rules differ in their capacity and the average size of the basins of attraction for memories at a given memory load $\alpha$, further discussed Section 3.4 of the Supplementary Material.

### Simulations

We simulate the memory storage, retrieval, and decoding processes by creating dictionaries of objects and attributes $\mathcal {D}^{a}$ and $\mathcal {D}^{b}$ where *a*’s and *b*’ s are random vectors with components drawn iid from a Gaussian distribution i.e. $a_{i}\sim \mathcal {N}(0,\frac{1}{N})$, $b_{i}\sim \mathcal {N}(0,\frac{1}{N})$. The size of these dictionaries is fixed to $D=L_{max}N$, where $L_{max}$ is the size of the largest structure being considered. In Fig. 1b we show the decoding error for several values of $L_{max}$ and for Figs. 1c and 3 we set $L_{max}=30$. We then use a subset of the dictionaries to create *P* knowledge structures with vector representations given by HRR encoding. These structures are then point-wise binarized and used to compute the synaptic weight matrix using the Pseudo-inverse rule unless otherwise stated. Since the encoding of the structures induces a similarity with individual relations *g*(*a*, *b*) rather than with *a* or *b* individually, we find that the same set of attributes $\lbrace b_{1},b_{2},\dots b_{L}\rbrace$ can be the same across several or all of the different knowledge structures while retaining the ability to decode the corresponding object $a_{\ell }^{\mu }$ from retrieved structure $\sigma _{r}^{\mu }$. Hence, we consider the case in which the same attributes are used in each structure i.e. $b_{\ell }^{\mu }=b_{\ell }$.

We test the performance of the memory network by initializing the network in the state $\sigma ^{\mu }(0)=\sigma _{0}^{\mu }=\text {sgn}(\widehat{S}_{0}^{\mu })$ for each memory $\mu =1,\dots ,P$ where $S_{0}^{\mu }=\lbrace (a_{\ell }^{\mu },b_{\ell }^{\mu })\rbrace _{\ell =1}^{L_{0}}$ is the subset of $L_{0}$ relations used to create a retrieval cue. We then evolve the network for *T* parallel updates, denoting the attractor reached by the network as $\sigma _{r}^{\mu }=\sigma ^{\mu }(T)$ , i.e., the retrieved state starting from partial cue of the $\mu$-th structure. We define $m^{\mu }$ as the overlap between $\sigma ^{\mu }$ and $\sigma _{r}^{\mu }$ i.e.

29 $$\begin{aligned} m^{\mu }&=\frac{1}{N}\sum _{i=1}^{N}\sigma _{i}^{\mu }\sigma ^{\mu }_{ri} \end{aligned}$$

For each retrieved structure $\sigma _{r}^{\mu }$, we use $b_{L}^{\mu }$, corresponding to a relation *not* contained in the initializing structure, to obtain an estimate $\hat{a}_{L}^{\mu }$ for $a_{L}^{\mu }$, which then identified with the Dictionary element with which it has the highest overlap.

The Pseudo-inverse rule ensures that the basins of attraction for different structures are essentially identical regardless of potential differences in the overlap between different structures. In simulations, this allows us to consider each structure as an independent trial. The fraction of trials in which $a_{L}^{\mu }$ is incorrectly decoded from $\sigma _{r}^{\mu }$ provides an empirical estimate of the decoding error $P_{\epsilon }$. We also construct an empirical SNR from Eq. (20). Finally, we measure $m^{\mu }$ for each structure (Eq. 29) to obtain an empirical distribution *p*(*m*) where the overlaps *m* are calculated for each memory in a trial and accumulated over many trials. The distribution *p*(*m*) does not appear to change if measured over multiple trials with different patterns or for multiple patterns within the same trial, which further supports the ability to consider each structure as a separate trial. The distribution *p*(*m*) is a statistical measure of the retrieval quality for structures of fixed size *L*, memory load $\alpha$, and retrieval cue length $L_{0}$.

### Determination of $l_{c}$

To determine $l_{c}$ as a function of the various network parameters, we calculate the relation between $L_{0}/L$ and the average initial overlap $m_{0}$ with the desired structure in the limit of large *N*, yielding

30 $$\begin{aligned} m_{0}&\approx \frac{2}{\pi }\arctan \left( \left( \frac{\frac{L_{0}}{L}}{1-\frac{L_{0}}{L}}\right) ^{\frac{1}{2}}\right) \end{aligned}$$

Further details of the derivation are provided in Supplementary Section 1.4 of the Supplementary Material. Using Eq. (30), we write $l_{c}(\alpha )$ in terms of $m_{min}(\alpha )$ defined in the main text as

31 $$\begin{aligned} l_{c}(\alpha )=\min \frac{L_{0}}{L}&\approx \frac{\tan ^{2}(\frac{\pi }{2}m_{min}^{2}(\alpha ))}{1+\tan ^{2}(\frac{\pi }{2}m{}_{min}^{2}(\alpha ))} \end{aligned}$$

To determine $m_{min}(\alpha )$ we resort to the Pseudo-inverse model with random binary patterns as memories^38^, which is simpler to simulate. Results relating $m_{min}(\alpha )$ and $l_{c}$ are shown in Supplementary Fig. 1.

### Empirical distribution of overlaps

We find that the empirical distribution *p*(*m*) is bimodal and takes the general form

32 $$\begin{aligned} p(m)&=(1-p_{1})p_{m<1}(m)+p_{1}\delta (m-1) \end{aligned}$$

where $p_{1}$ is the probability that a structure is perfectly retrieved from memory and $p_{m<1}(m)$ corresponds to the distribution of *m* for imperfectly retrieved memories.

The peak at $m=1$ corresponds to trajectories converging to the target memories. This can be nonzero even when initial overlap $m_{0}$ is outside the mean basin radius, indicating non-spherical basin shape. The second mode, peaked at $0<m<1$ results from trajectories that converged to a fixed point outside the basin with a significant residual overlap with the memory. We characterize the shape of the distribution by the probability of $m=1$, $p_1$, the width of the lower *m* mode, $\sigma _{m}$ and the mean of that mode, $m^{*}$. Results are shown in Supplementary Fig. 4a, for several values of *N* and two values of $\alpha$.

As noted in^38,40^, the shape of the distribution *p*(*m*) is sensitive to finite size effects. To analyze these effects, we calculate *p*(*m*) for different sizes in a standard Pseudo-inverse model where the initial overlap $m_{0}$ can essentially be varied continuously. For $m_{0}>m_{min}(\alpha )$ almost all trials converge to the memorized pattern. For a range of values $m_{0}<m_{min}(\alpha )$, *p*(*m*) is bimodal. We find that *p*(*m*) obtained from networks storing random patterns is very similar to the distribution obtain from networks storing structure memories, when the $m_{0}$ and $L_{0}/L$ are related as in Eq. (30). We find that for large *N*, $p_{1}$ approaches a step function changing from zero to one as $m_0$ crosses $m_{min}(\alpha )=1-R(\alpha )$. Near this transition, $p_{1}$ can be approximated as

33 $$\begin{aligned} p_{1}&\sim \frac{1}{1+e^{-\sqrt{N}(m_{0}-m_{min}(\alpha ))}} \end{aligned}$$

indicating that it converges to a step function exponentially fast with $\sqrt{N}$. In addition, $\sigma _{m}$ is very small outside the narrow transition regime of $m_{0}$ and shrinks to zero everywhere as $1/\sqrt{N}$. From this, we conclude that for $N\rightarrow \infty$, *p*(*m*) becomes a $\delta$ function, which is either located at $m=1$ for $m_0>m_{min}$ or at a smaller value $m^{*}$ which increases smoothly with $m_0$, starting from zero and reaching 1 as $m_0$ increases from zero to $m_{min}$. Thus in large networks, the basins have a roughly spherical shape, such that virtually all initial conditions with $m_{0}\ge m_{min}$ converge to the memory, and all initial conditions with $m_{0}<m_{min}$ converge to fixed points with partial overlap, $m^{*}$.

## Supplementary Information

Supplementary Information.

## Data Availability

The data that support the findings of this study are available from the corresponding authors upon reasonable request.
