# Supplementary material for: Associative memory of structured knowledge
Source: Sci Rep. 2022 Dec 17;12:21808. doi: 10.1038/s41598-022-25708-y (PMC9759586; doi:10.1038/s41598-022-25708-y)
Supplement: Supplementary file 1 — Supplementary Information. [file 41598_2022_25708_MOESM1_ESM.pdf]

Supplementary Material for Associative memory of structured knowledge

Julia Steinberg[1,2,3,*] **and Haim Sompolinsky**[2,4]

[1]Department of Physics, Harvard University, Cambridge, Massachusetts 02138, USA
[2]Center for Brain Science, Harvard University, Cambridge MA 02138, USA
[3]Joseph Henry Laboratories of Physics, Princeton University, Princeton NJ 08544, USA
[4]Edmond and Lily Safra Center for Brain Sciences, Hebrew University, Jerusalem 91904, Israel
[*]jasteinberg@alumni.harvard.edu
[+]haim@fiz.huji.ac.il

ABSTRACT

Contents

1 Holographic Reduced Representation **1**
1.1 Overview . . . 1
1.2 Clean-up and SNR . . . 2
1.3 Binarization and Empirical SNR . . . 4
1.4 Memory Initialization . . . 5
2 Decoding Error **6**
2.1 Limitations for Good Decoding . . . 7
2.2 Decoding After Memory Retrieval . . . 8
3 Insights from Random Patterns **9**
3.1 Network Order Parameters . . . 9
3.2 Overlap Distributions . . . 9
3.3 Retrieval Dynamics . . . 9
3.4 Comparing Learning Rules . . . 10
3.5 Connection to Storage of Knowledge Structures . . . 14
4 Temporal Sequences as Sequences of Attractors **16**
References **17**

1 Holographic Reduced Representation

1.1 Overview

In holographic reduced representation (HRR)[1], the binding operation g is circular convolution

$$g = \text{cconv}(a, b) \tag{1}$$

where the individual components of g are given by

$$g_j = \sum_{k=0}^{N-1} a_k b_{j-k} = \sum_{k=0}^{N-1}\sum_{m=0}^{N-1} \delta_{j,k+m} a_k b_m \;,\; j = 0, \ldots, N-1 \tag{2}$$

with all subscripts are defined modulo N. For objects and attributes $a, b \in \mathbb{R}^N$, this creates a representation $g(a,b) \in \mathbb{R}^N$. The circular convolution operation is both associative and commutative, i.e. $\text{cconv}(a,b) = \text{cconv}(b,a)$.

The corresponding decoding operation is given by the circular correlation

$$f = \mathrm{ccorr}(\widehat{S}, b) \tag{3}$$

where the individual components of f are given by

$$f_j = \sum_{k=0}^{N-1} \widehat{S}_k b_{j+k,\ell} = \sum_{k=0}^{N-1}\sum_{m=0}^{N-1} \delta_{j,m-k} \widehat{S}_k b_m \ , \ j = 0, \ldots, N-1 \tag{4}$$

In general the circular correlation operation is neither associative nor commutative. However, it can be related to circular convolution by defining the involution of a vector a as a^* where $a^*_j = a_{-j}$. Then $\mathrm{ccorr}(a,b) = \mathrm{cconv}(a^*, b)$ and a^* as serves as an approximate inverse to a when both a and b are random vectors.

1.2 Clean-up and SNR

To quantify the effect of the ML clean-up operation described in the main text, we compute various statistics of the estimator $\hat{a}_\ell$ for the corresponding object a_ℓ decoded from a relational structure $\widehat{S}$ using the HRR unbinding operation. We consider the case where all objects and attributes are random vectors with components drawn iid, i.e., $a_{id} \sim \mathcal{N}(0, \frac{1}{N})$, $b_{id} \sim \mathcal{N}(0, \frac{1}{N})$ and decoding is done from a dictionary of size D.

Since the encoding operation is permutation invariant, the statistics for all $\hat{a}_\ell$ decoded from the full structure should be the same. For simplicity we set $\ell = 1$ and consider $\hat{a}_1$. We index dictionary items so that indices $\ell < L$ correspond to the dictionary items contained in the structure $\widehat{S}$ and indices $L < \ell < D$ corresponds to dictionary items that are not contained in $\widehat{S}$. Note that

$$\langle a_{i,\ell} a_{j,\ell'} \rangle = \frac{1}{N} \delta_{ij} \delta_{\ell\ell'} \tag{5}$$

$$\langle a_{i,\ell} a_{j,\ell'} a_{k,s} a_{l,s'} \rangle = \frac{1}{N^2} (\delta_{ij}\delta_{\ell\ell'}\delta_{kl}\delta_{ss'} + \delta_{ik}\delta_{\ell s}\delta_{jl}\delta_{\ell'}\delta_{ss'} + \delta_{il}\delta_{\ell s'}\delta_{jk}\delta_{\ell' s}) \tag{6}$$

Using the expression for the unbinding operation in Eqn. 4, we can express the estimator $\hat{a}_1$ as

$$\hat{a}_{j,1} = \sum_{k=0}^{N-1} \widehat{S}_k b_{j+k,1} \tag{7}$$

Using Eqn. 2, we can express this in terms of a_ℓ's and a_ℓ's, and $\hat{a}_{j,1}$ as

$$\begin{aligned} \hat{a}_{j,1} &= a_{j,1} \sum_{k=0}^{N-1} \left(b_{k,1}\right)^2 + \sum_{k=0,k'\neq k}^{N-1} a_{k-k'+j,1} b_{k,1} b_{k',1} + \sum_{\ell=2}^{L}\sum_{k=0}^{N-1}\sum_{k'=0}^{N-1} a_{k-k'+j,\ell} b_{k,\ell} b_{k',1} \\ &= (1+\varepsilon)\, a_{j,1} + \xi_{j,1} + \xi_{j,2} \end{aligned} \tag{8}$$

where we have defined the three noise terms as

$$\varepsilon = \sum_{k=0}^{N-1} \left(b_{k,1}\right)^2 - 1 \tag{9}$$

$$\xi_{j,1} = \sum_{k=0}^{N-1}\sum_{k'=0}^{N-1} (1 - \delta_{kk'}) b_{k,1} b_{k',1} a_{k-k'+j,1} \tag{10}$$

$$\xi_{j,2} = \sum_{\ell=2}^{L}\sum_{k=0}^{N-1}\sum_{k'=0}^{N-1} b_{k,1} b_{k',\ell} a_{k-k'+j,\ell} \tag{11}$$

Here, ε is noise due to deviations in the normalization of a, $\xi_{j,1}$ is noise from interference elements of different elements of the cue within the same attribute b_1, and $\xi_{j,2}$ and $\xi_{j,2}$ are noise coming from interference between attribute b_1 with all other attributes b_ℓ contained in the structure. Since components of a_1 appear an odd number of times in each term of the sums in $\xi_{j,1}$ and $\xi_{j,2}$, we conclude that $\langle \xi_{j,1} \rangle = \langle \xi_{j,2} \rangle = 0$. Likewise, $\langle \varepsilon \rangle = 0$. Using Eqns. 5 and 6, we find that the second moments and

correlation of the two noise terms are given by

$$\begin{aligned}
\langle \varepsilon^2 \rangle &= 1 + \left(\sum_{k=0}^{N-1} \sum_{q=0}^{N-1} \langle b_k^2 b_q^2 \rangle \right) - 2 \sum_{k=0}^{N-1} \langle b_k^2 \rangle \qquad (12) \\
&= \sum_{k=0}^{N-1} \sum_{q=0}^{N-1} \frac{1}{N^2} (1 + 2\delta_{kq}) - 1 \\
&= \frac{2}{N} \\
\langle \xi_{j,1}^2 \rangle &= \sum_{k=0}^{N-1} \sum_{k'=0}^{N-1} \sum_{q=0}^{N-1} \sum_{q'=0}^{N-1} (1 - \delta_{kk'})(1 - \delta_{qq'}) \langle b_{k,1} b_{k',1} b_{q,1} b_{q',1} \rangle \langle a_{k-k'+j,1} a_{q-q'+j,1} \rangle \qquad (13) \\
&= \sum_{k=0}^{N-1} \sum_{k'=0}^{N-1} \sum_{q=0}^{N-1} \sum_{q'=0}^{N-1} \frac{1}{N^3} (\delta_{kq} \delta_{k'q'} - \delta_{kk'} \delta_{qq'} \delta_{kq}) \\
&= \frac{(N-1)}{N^2} \\
\langle \xi_{j,2}^2 \rangle &= \sum_{\ell=2}^{L} \sum_{\ell'=2}^{L} \sum_{k=0}^{N-1} \sum_{k'=0}^{N-1} \sum_{q=0}^{N-1} \sum_{q'=0}^{N-1} \langle b_{k,1} b_{k',\ell} b_{q,1} b_{q',\ell'} \rangle \langle a_{k-k'+j,\ell} a_{q-q'+j,\ell'} \rangle \qquad (14) \\
&= \sum_{\ell=2}^{L} \sum_{\ell'=2}^{L} \sum_{k=0}^{N-1} \sum_{k'=0}^{N-1} \sum_{q=0}^{N-1} \sum_{q'=0}^{N-1} \frac{1}{N^3} (\delta_{1\ell} \delta_{\ell\ell'} \delta_{kk'} \delta_{qq'} + \delta_{\ell\ell'} \delta_{kq} \delta_{k'q'} + \delta_{1\ell} \delta_{\ell\ell'} \delta_{kq'} \delta_{k'q} \delta_{k-k',q-q'}) \\
&= \frac{L}{N} + \frac{1}{N^2} \\
\langle \xi_{j,1} \xi_{j,2} \rangle &= \sum_{\ell=2}^{L} \sum_{k=0}^{N-1} \sum_{k'=0}^{N-1} \sum_{q=0}^{N-1} \sum_{q'=0}^{N-1} (1 - \delta_{kk'}) \langle b_{k,1} b_{k',1} b_{q,1} b_{q',\ell} \rangle \langle a_{k-k'+j,1} a_{q-q'+j,\ell} \rangle \qquad (15) \\
&= 0
\end{aligned}$$

and from Eqn. 8, we see the noise in each component of the estimator is

$$\frac{\langle \delta \hat{a}_{j,\ell} \rangle}{\langle a_{j,\ell}^2 \rangle} \sim L \qquad (16)$$

We now calculate the first two moments of the overlap of the estimator with the correct pattern $a_d \cdot \hat{a}_1$ to obtain the SNR for HRR defined in Eqn. 20 of Methods. $a_d \cdot \hat{a}_1$ can be expressed as

$$a_d \cdot \hat{a}_1 = (1 + \varepsilon) a_d \cdot a_1 + a_d \cdot (\xi_1 + \xi_2) \qquad (17)$$

We see from Eqn. 17 that $\langle a_1 \cdot \hat{a}_1 \rangle = 1$ and $\langle a_d \cdot \hat{a}_1 \rangle = 0$, so the estimator is unbiased. The second moment is given by

$$\begin{aligned}
\langle (a_d \cdot \hat{a}_1)^2 \rangle &= \left\langle \sum_{j=0}^{N-1} \sum_{j'=0}^{N-1} \sum_{k=0}^{N-1} \sum_{k'=0}^{N-1} \sum_{q=0}^{N-1} \sum_{q'=0}^{N-1} \sum_{\ell=1}^{L} \sum_{\ell'=1}^{L} b_{k,1} b_{k',\ell} a_{k-k'+j,\ell} a_{j,d} b_{q,1} b_{q',\ell'} a_{q-q'+j',\ell'} a_{j',d} \right\rangle \qquad (18) \\
&= \sum_{j=0}^{N-1} \sum_{j'=0}^{N-1} \sum_{k=0}^{N-1} \sum_{k'=0}^{N-1} \sum_{q=0}^{N-1} \sum_{q'=0}^{N-1} \sum_{\ell=1}^{L} \sum_{\ell'=1}^{L} \langle b_{k,1} b_{k',\ell} b_{q,1} b_{q',\ell'} \rangle \langle a_{k-k'+j,\ell} a_{j,d} a_{q-q'+j',\ell'} a_{j',d} \rangle \\
&= \sum_{j=0}^{N-1} \sum_{j'=0}^{N-1} \sum_{k=0}^{N-1} \sum_{k'=0}^{N-1} \sum_{q=0}^{N-1} \sum_{q'=0}^{N-1} \sum_{\ell=1}^{L} \sum_{\ell'=1}^{L} \frac{1}{N^4} (\delta_{kk'} \delta_{qq'} \delta_{d\ell} \delta_{1\ell'} \delta_{\ell\ell'} + \delta_{kk'} \delta_{qq'} \delta_{kq} \delta_{\ell\ell'} \delta_{1\ell} + \delta_{kk'} \delta_{qq'} \delta_{jj'} \delta_{d\ell} \delta_{1\ell'} \delta_{\ell\ell'} \\
&+ \delta_{kk'} \delta_{qq'} \delta_{kq} \delta_{\ell\ell'} \delta_{\ell d} + \delta_{kq} \delta_{k'q'} \delta_{jj'} \delta_{\ell\ell'} + \delta_{kq} \delta_{k'q'} \delta_{jj'} \delta_{\ell\ell'} \delta_{\ell d} + \delta_{kk'} \delta_{qq'} \delta_{kq} \delta_{1\ell'} \delta_{d\ell} \delta_{\ell\ell'} + \delta_{kk'} \delta_{qq'} \delta_{kq} \delta_{jj'} \delta_{\ell\ell'} \delta_{1\ell} \\
&+ \delta_{kk'} \delta_{qq'} \delta_{kq} \delta_{jj'} \delta_{1\ell} \delta_{d\ell'} \delta_{\ell\ell'}) \\
&= \delta_{1d} + \frac{L}{N} + \frac{1}{N} (1 + 2\delta_{1d} + 2\Theta(L-d)) + \frac{1}{N^2} (1 + \delta_{1d}))
\end{aligned}$$

To summarize

$$\langle a_d \cdot \hat{a}_1 \rangle = \begin{cases} 1 & d = 1 \\ 0 & 1 < d \leq L \\ 0 & L < d \end{cases} \tag{19}$$

$$\Sigma_{1d}^2 = \langle (a_d \cdot \hat{a}_1 - \langle a_d \cdot \hat{a}_1 \rangle)^2 \rangle = \begin{cases} \frac{L+5}{N} + \frac{2}{N^2} & d = 1 \\ \frac{L+3}{N} + \frac{1}{N^2} & 1 < d \\ \frac{L+1}{N} + \frac{1}{N^2} & L < d \end{cases} \tag{20}$$

For $L \ll N$, the SNR for overlaps defined in Eqn. 20 of the main text is then approximately

$$\frac{\langle a_1 \cdot \hat{a}_1 \rangle^2}{\langle (a_d \cdot \hat{a}_1)^2 \rangle} \approx \frac{N}{L} \tag{21}$$

1.3 Binarization and Empirical SNR

When decoding from a binarized structure $\sigma = \text{sgn}(\widehat{S})$, the estimator $\hat{a}_1$ is given by

$$\hat{a}_{j1} = \sum_{k=0}^{N-1} \sigma_k b_{k+j,1} \tag{22}$$

where

$$\sigma_j = \text{sgn}\left(\sum_{k=0}^{N-1} \sum_{\ell=1}^{L} a_{k,\ell} b_{j-k,\ell} \right) \tag{23}$$

To compute the SNR, we start by evaluating the overlap between the estimator $\hat{a}_1$ with items in the dictionary $\langle a_d \cdot \hat{a}_1 \rangle$ which can be expressed as

$$\langle a_d \cdot \hat{a}_1 \rangle = \left\langle \sum_{j=0}^{N-1} \sum_{k=0}^{N-1} a_{j,d} b_{k,1} \text{sgn}\left(a_{j,1} b_{k,1} + \sum_{q \neq k}^{N-1} a_{k-q+j,1} b_{q,1} + \sum_{\ell=2}^{L} \sum_{q=0}^{N-1} a_{k-q+j,\ell} b_{q,\ell} \right) \right\rangle \tag{24}$$

While individual products of elements $a_{\ell,i} b_{\ell,j}$ are not Gaussian distributed, the expectation $\langle a_d \cdot \hat{a}_1 \rangle$ is the sum of N^2 random variables with correlations only occurring at higher order as the expectation of the product of different elements of each circular convolution is zero. Since all terms contained in the sums over indiced j and k in Eqn. 24 are only correlated at higher order, we can approximate each term in the sum as independent which gives us

$$\langle a_d \cdot \hat{a}_1 \rangle \approx N^2 \left\langle a_{0,d} b_{0,1} \left(\text{sgn}\left(a_{0,1} b_{0,1} + \sum_{q=1}^{N-1} a_{N-q,1} b_{q,1} + \sum_{\ell=2}^{L} \sum_{q=0}^{N-1} a_{N-q,\ell} b_{q,\ell} \right) \right) \right\rangle \tag{25}$$

We now define the following three random variables

$$z_1 = a_{0,1} b_{0,1} \tag{26}$$

$$z_d = a_{0,d} b_{0,1} \tag{27}$$

$$\xi = \sum_{q=1}^{N-1} a_{N-q,1} b_{q,1} + \sum_{\ell=2}^{L} \sum_{q=0}^{N-1} a_{N-q,\ell} b_{q,\ell} \tag{28}$$

where x_1 is a signal term and ξ is a noise term within the sign function. $\langle z_1 \rangle = \langle z_2 \rangle = \langle \xi \rangle = 0$ and the variances are given by

$$\Sigma_{z_1}^2 = \frac{1}{N^2} \tag{29}$$

$$\Sigma_{z_\ell}^2 = \frac{1}{N^2} \tag{30}$$

$$\Sigma_{\xi}^2 = \frac{(N-1)}{N^2} + \frac{L}{N} \sim \frac{L}{N} \tag{31}$$

For $d = 1$, $z_1 = z_\ell = z$ and $L \gg 1$ we can treat z and ξ as Gaussian. In this approximation $\langle a_1 \cdot \hat{a}_1 \rangle$ becomes

$$\begin{aligned}\langle a_1 \cdot \hat{a}_1 \rangle &\approx N^2 \int_{-\infty}^{\infty} \frac{dz}{\sqrt{2\pi\Sigma_z^2}} \int_{-\infty}^{\infty} \frac{d\xi}{\sqrt{2\pi\Sigma_\xi^2}} e^{-\frac{z^2}{2\Sigma_z^2} - \frac{\xi^2}{2\Sigma_\xi^2}} z \operatorname{sgn}(z+\xi) \\ &= \sqrt{\frac{2}{\pi}} \frac{N^2 \Sigma_z^2}{\sqrt{\Sigma_z^2 + \Sigma_\xi^2}} \\ &\sim \sqrt{\frac{2N}{\pi L}}\end{aligned} \tag{32}$$

Likewise, the second moment is given by

$$\langle (a_1 \cdot \hat{a}_1)^2 \rangle = \langle (a_d \cdot \hat{a}_1)^2 \rangle \approx N^2 \int_{-\infty}^{\infty} \frac{dz}{\sqrt{2\pi\Sigma_z^2}} e^{-\frac{z^2}{2\Sigma}} \int_{-\infty}^{\infty} \frac{d\xi}{\sqrt{2\pi\Sigma_\xi^2}} e^{-\frac{\xi^2}{2\Sigma_\xi^2}} z^2 = 1 \tag{33}$$

Putting Eqns. 32 and 33 together, the SNR of overlap when decoding a_1 from the binarized structure σ is given by

$$\mathrm{SNR} = \frac{\langle a_1 \cdot \hat{a}_1 \rangle^2}{\langle (a_d \cdot \hat{a}_1)^2 \rangle} \sim \frac{2}{\pi} \frac{N}{L} \tag{34}$$

We see from Eqn. 21 that binarizing $\widehat{S}$ has decreased the SNR by a constant factor of $\frac{2}{\pi}$.

1.4 Memory Initialization

We now determine the average initial overlap m_0 between a binarized retrieval structure σ_0 containing L_0 of the L relations in the binarized full structure σ in the case of random a_ℓ's and b_ℓ's are random vectors with components drawn iid as before. An expression for the average overlap m_0 between σ_0 and σ can be written as

$$m_0 = \left\langle \frac{1}{N} \sum_{j=0}^{N-1} \operatorname{sgn}\left(\widehat{S}_j\right) \operatorname{sgn}\left(\widehat{S}_{j,0}\right) \right\rangle \tag{35}$$

The overlap between components of the unbinarized structures $\widehat{S}_j$ and $\widehat{S}_{j'0}$ with HRR binding is given by

$$\begin{aligned}\langle \widehat{S}_j \widehat{S}_{j'0} \rangle &= \sum_{\ell=1}^{L} \sum_{\ell'=1}^{L_0} \sum_{k=0}^{N-1} \sum_{k'=0}^{N-1} \sum_{p=0}^{N-1} \sum_{p'=0}^{N-1} \delta_{p',j'-k'} \delta_{p,j-k} a_{k,\ell} a_{k',\ell'} b_{p,\ell} b_{p',\ell'} \Big\rangle \\ &= \left\langle \sum_{\ell=1}^{L_0} \sum_{k=0}^{N-1} \delta_{jj'} a_{k,\ell}^2 b_{j-k,\ell}^2 \right\rangle \\ &= \frac{L_0}{N} \delta_{jj'}\end{aligned} \tag{36}$$

For large N we can approximate the sum in Eqn. 35 by treating the individual terms as as independent since there are no lower order correlations between terms. We can then define the random variables $z_1 = \widehat{S}_{j0}$ and $z_2 = \widehat{S}_j - \widehat{S}_{j0}$ as approximately Gaussian distributed with zero mean and variances $\Sigma_{z_1}^2 = \frac{L_0}{N}$ and $\Sigma_{z_2}^2 = \frac{(L-L_0)}{N}$ respectively. Then, Eqn. 35 can be approximated as

$$\begin{aligned}m_0 &\approx \int_{-\infty}^{\infty} \frac{dz_1}{\sqrt{\Sigma_{z_1}^2}} e^{-\frac{z_1^2}{2\Sigma_{z_1}^2}} \int_{-\infty}^{\infty} \frac{dz_2}{\sqrt{2\pi\Sigma_{z_2}^2}} e^{-\frac{z_2^2}{2\Sigma_{z_2}^2}} \operatorname{sgn}(z_1)\operatorname{sgn}(z_1+z_2) \\ &\approx \frac{2}{\pi} \arctan\left(\frac{\Sigma_{z_1}}{\Sigma_{z_2}}\right) \\ &\approx \frac{2}{\pi} \arctan\left(\left(\frac{\frac{L_0}{L}}{1-\frac{L_0}{L}}\right)^{\frac{1}{2}}\right)\end{aligned} \tag{37}$$

For $L_0 \ll L$ Eqn. 37 can be further approximated as

$$m_0 \approx \frac{2}{\pi}\sqrt{\frac{L_0}{L}} \quad (38)$$

This implies that creating retrieval structures with L_0 out of L relations will result in retrieval cues with average overlap m_0 with the full memorized structure. The variance of the distribution of initial overlaps will vanish as $N \to \infty$.

We use this relation to determine $l_c(\alpha)$ as a function of L_0/L using the value of $m_{min}(\alpha)$ obtained for a Pseudo-inverse model with random binary patterns as memories[2]. Results for $m_{min}(\alpha)$ are shown in Fig. 1a demonstrating a linear increase in the range $\alpha < 0.5$. Substituting these results in Eqn. 31 closely predicts the value of $l_c(\alpha)$ as shown in Fig. 1b.

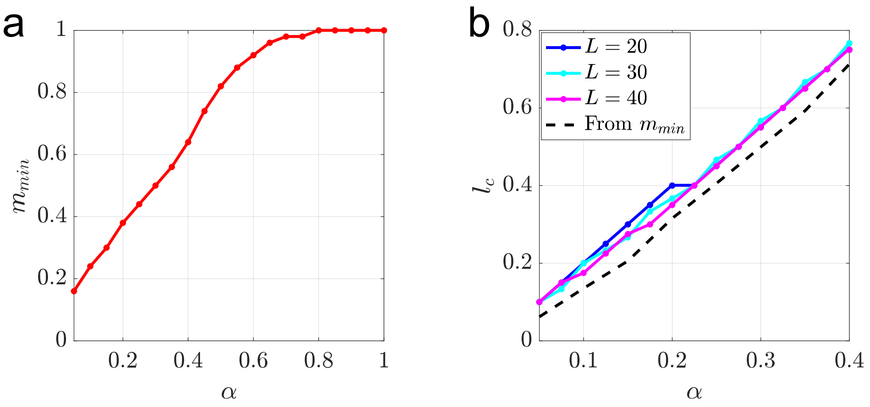

Figure 1. (a) m_{min} shown as a function of α for a Pseudo-inverse network storing with random patterns. (b) For structured memories, m_{min} corresponds to a minimum fraction of relations needed obtain a final overlap $m = 1$, shown for $L = 20, 30, 40$ along with the value predicted by m_{min} from the expression in Eqn. 31. $N = 1000$ and $T = 20$ parallel updates are used in memory retrieval for both figures and the averages are computed over 50 trials.

2 Decoding Error

We derive a good approximation for the ML decoding error in terms of D, the size of the decoding dictionary, and SNR, the signal-to-noise ratio of overlaps defined in Eqn. 20 of Methods. The overlap $\mathscr{O}_{d1}$ between a dictionary item a_d with the estimator $\hat{a}_1$ for a_1 decoded from a structure $\widehat{S}$ is given by

$$\mathscr{O}_{1d} = \hat{a}_1 \cdot a_d \quad (39)$$

where as in the previous section, we index items so that $d \leq L$ corresponds to overlap with patterns within $\widehat{S}$ and $L < d$ corresponds to overlaps with other items in the Dictionary not contained in $\widehat{S}$. For ML decoding, an error occurs if $\max(\mathscr{O}_{12}, \ldots, \mathscr{O}_{1D}) \geq \mathscr{O}_{11}$. The probability of error P_ε is then given by

$$P_\varepsilon = 1 - \int_{-\infty}^{\infty} d\mathscr{O}_{11} P(\mathscr{O}_{11}) \left(\int_{-\infty}^{\mathscr{O}_{11}} d\mathscr{O}_{1L} P(\mathscr{O}_{1L})\right)^{L-1} \left(\int_{-\infty}^{\mathscr{O}_{11}} d\mathscr{O}_{1D} P(\mathscr{O}_{1D})\right)^{D-L} \quad (40)$$

For large N, each $\mathscr{O}_{1d}$ essentially behaves as an independent Gaussian random variable with mean μ_{1d} and variance Σ_{1d}. For unbiased decoding schemes, $\mu_{1L} = \mu_{1D} = 0$. In general, we can consider decoding from a structure containing errors by setting $\mu_{11} = \mu$, where μ is related to the overlap of the corrupted structure with the correct structure. Then P_ε can be very well approximated as

$$P_\varepsilon \approx \int_{-\infty}^{\infty} Dz \left(1 - H\left(-\frac{\Sigma_{11} z + \mu}{\Sigma_{1L}}\right)^{L-1} H\left(-\frac{\Sigma_{11} z + \mu}{\Sigma_{1D}}\right)^{D-L}\right) \quad (41)$$

where $Dz = \frac{dz}{\sqrt{2\pi}} e^{-\frac{z^2}{2}}$.

For HRR $\Sigma_{11} \approx \Sigma_{1L} \approx \Sigma_{1D}$ to leading order in $\frac{1}{N}$. Defining Σ to be the leading order term in the variance, we obtain

$$P_\varepsilon \approx \int_{-\infty}^{\infty} Dz \left(1 - H\left(-z - \frac{\mu}{\Sigma}\right)^D\right) \tag{42}$$

Identifying $\frac{\mu^2}{\Sigma^2}$ with the SNR of overlaps, Eqn. 42 becomes

$$P_\varepsilon \approx \int_{-\infty}^{\infty} Dz \left(1 - H\left(-z - \sqrt{\mathrm{SNR}}\right)^D\right) \tag{43}$$

given in the main text.

2.1 Limitations for Good Decoding

We can understand the dependence of P_ε on the SNR and D in the large SNR regime by obtaining a saddlepoint approximation for the expression for P_ε given in Eqn. 43. We start by rewriting Eqn. 43 as

$$P_\varepsilon \approx \int_{-\infty}^{\infty} \frac{dz}{\sqrt{2\pi}} e^{f(z)} \tag{44}$$

where $f(z)$ is given by

$$f(z) = -\frac{z^2}{2} + \log\left(1 - H\left(-z - \sqrt{\mathrm{SNR}}\right)^D\right) \tag{45}$$

From Eqn. 42, we see $P_\varepsilon \ll 1$ for $H(z+\sqrt{\mathrm{SNR}}) \ll 1$ as $H(-z-\sqrt{\mathrm{SNR}}) = 1 - H(z+\sqrt{\mathrm{SNR}})$. So we are interested in the regime where $1 \ll z + \sqrt{\mathrm{SNR}}$. In this regime, $f(z)$ is very large, so the integral in P_ε can be approximated its saddlepoint value

$$P_\varepsilon \approx \frac{1}{\sqrt{|f''(z_0)|}} e^{f(z_0)} \tag{46}$$

where z_0 is given by the solution of

$$z = -\frac{DH\left(-z-\sqrt{\mathrm{SNR}}\right)^{D-1}}{1 - H\left(-z-\sqrt{\mathrm{SNR}}\right)^D} \frac{e^{-\frac{(z+\sqrt{\mathrm{SNR}})^2}{2}}}{\sqrt{2\pi}} \tag{47}$$

In this regime we can make the approximation

$$H\left(-z-\sqrt{\mathrm{SNR}}\right)^{-D} \approx e^{DH\left(z+\sqrt{\mathrm{SNR}}\right)} \tag{48}$$

which allows us to approximate Eqn. 47 as

$$\begin{aligned} z &= -\frac{DH\left(-z-\sqrt{\mathrm{SNR}}\right)^{D-1}}{1 - H\left(-z-\sqrt{\mathrm{SNR}}\right)^D} \frac{e^{-\frac{(z+\sqrt{\mathrm{SNR}})^2}{2}}}{\sqrt{2\pi}} \\ &\approx -\frac{DH\left(z+\sqrt{\mathrm{SNR}}\right)}{H\left(-z-\sqrt{\mathrm{SNR}}\right)^{-D} - 1}\left(z+\sqrt{\mathrm{SNR}}\right) \\ &\approx -\frac{DH\left(z+\sqrt{\mathrm{SNR}}\right)}{e^{DH\left(z+\sqrt{\mathrm{SNR}}\right)} - 1}\left(z+\sqrt{\mathrm{SNR}}\right) \end{aligned} \tag{49}$$

where for $z + \sqrt{\mathrm{SNR}} \gg 1$ we used

$$H\left(z+\sqrt{\mathrm{SNR}}\right) \approx \frac{e^{-\frac{1}{2}(z+\sqrt{\mathrm{SNR}})^2}}{\sqrt{2\pi}\left(z+\sqrt{\mathrm{SNR}}\right)} \tag{50}$$

From Eqn. 49 we see that for $DH\left(z+\sqrt{\mathrm{SNR}}\right) \ll 1$, $z \to -\frac{\sqrt{\mathrm{SNR}}}{2}$ and $f''(-\frac{\sqrt{\mathrm{SNR}}}{2}) \approx -1$. The saddlepoint approximation for the error is then

$$\begin{aligned} P_\varepsilon &\approx \int_{-\infty}^{\infty} Dz \left(1 - H\left(-z - \sqrt{\mathrm{SNR}}\right)^D\right) && (51)\\ &\approx \int_{-\infty}^{\infty} Dz e^{\log\left(1 - e^{-DH\left(z+\sqrt{\mathrm{SNR}}\right)}\right)} && (52)\\ &\approx e^{-\frac{\mathrm{SNR}}{8}} \left(1 - e^{-\sqrt{\frac{2}{\pi \mathrm{SNR}}} D e^{-\frac{\mathrm{SNR}}{8}}}\right) \end{aligned}$$

This can be further approximated as

$$P_\varepsilon \approx \sqrt{\frac{2}{\pi \mathrm{SNR}}} D e^{-\frac{\mathrm{SNR}}{4}} \tag{53}$$

We can insert the SNR of overlaps into Eqn. 53 to determine the limits on the size of D for good decoding. For HRR the approximation for the error gives us

$$P_\varepsilon \approx \sqrt{\frac{2L}{\pi N}} D e^{-\frac{N}{4L}} \tag{54}$$

which implies that for an error of $\delta \ll 1$

$$\frac{N}{L} \approx 4 \log\left(\frac{D}{\delta\sqrt{\pi}}\right) - 2\log 2 \log\left(\frac{D}{\delta\sqrt{\pi}}\right) \tag{55}$$

From this, we expect the error to be small as long and SNR is large and D is polynomial, and not exponential, in N.

2.2 Decoding After Memory Retrieval

To analyze the effect of memory retrieval on the decoding error, we start by considering decoding from a degraded binarized structure which is a Hamming distance $\frac{m-1}{2}$ from the uncorrupted binarized structure. In this case, the effective SNR is found by making the replacement $\langle a_1 \cdot \hat{a}_1 \rangle \to m$ in Eqn. 34. From this, we see that $\mathrm{SNR}(m)$ should take the form

$$\mathrm{SNR}(m) = \frac{2}{\pi}\left(\frac{m}{\Sigma}\right)^2 \sim \frac{2m^2}{\pi}\frac{N}{L} \tag{56}$$

When $\hat{a}_1$ is decoded from an imperfectly retrieved structured from memory, we can instead make the replacement $\langle a_1 \cdot \hat{a}_1 \rangle \to \sqrt{c} m$. Here, $c \sim O(1)$ is a constant factor accounting for differences in overlap of the structure with relations within and outside the retrieval cue. Then $\mathrm{SNR}(m)$ takes the modified form

$$\mathrm{SNR}(m) \sim \frac{2cm^2}{\pi}\frac{N}{L} \tag{57}$$

The decoding error after retrieval from memory is then given by

$$\begin{aligned} P_\varepsilon &\approx \int dm p(m) \int_{-\infty}^{\infty} Dz \left(1 - H\left(-z - \sqrt{\mathrm{SNR}(m)}\right)^D\right) && (58)\\ &\approx \int dm p(m) \int_{-\infty}^{\infty} Dz \left(1 - H\left(-z - \sqrt{\frac{2cm^2}{\pi}\frac{N}{L}}\right)^D\right) \end{aligned}$$

where $p(m)$ takes the same form as in Methods. For $N \to \infty$, $p(m) \to \delta(m - m^*)$ and the decoding error approaches

$$P_\varepsilon \approx \int_{-\infty}^{\infty} Dz \left(1 - H\left(-z - \sqrt{\frac{2cm^{*2}}{\pi}\frac{N}{L}}\right)^D\right) \tag{59}$$

3 Insights from Random Patterns

3.1 Network Order Parameters

For generic Hopfield networks, we can characterize the quality of memory retrieval by formally defining three network order parameters which quantify the overlap of the network state with the stored memories at each time step. The first is

$$m^{\mu}(t) = \frac{1}{N}\sum_{i=1}^{N}\sigma_i^{\mu}\sigma_i(t) \tag{60}$$

which represents the overlap of the network state $\sigma(t)$ with the corresponding pattern σ^{μ} at time t. If the initial state of the network has an $O(1)$ with a small number of patterns, i.e., $m_0 = m^{\mu}(0)$, the memory retrieval process can be sufficiently described by including an additional order parameter

$$r^{\mu}(t) = \frac{1}{\alpha}\sum_{\nu\neq\mu}^{P} m^{\nu}(t)^2 \tag{61}$$

which represents the overlap of the state with all patterns except for μ. We can also define

$$m^{\mu 0}(t) = \frac{1}{N}\sum_{i=1}^{N}\sigma_i^{\mu}(0)\sigma_i(t) \tag{62}$$

as the overlap of the state at time t with the initial state of the network after attempting to retrieve σ^{μ} where the overlap between $\sigma^{\mu}(0)$ and σ^{μ} is m_0. While m^0 is typically not considered for random patterns, it becomes relevant in the case of structured knowledge, where the retrieval cue can be constructed from a subset of relations rather than simply a corrupted version of a memory.

For large N, the distributions of order parameters over fixed retrieval conditions, i.e., $p(m)$, $p(r)$ and $p(m^0)$, are sharply peaked at the average values, denoted by $\langle m\rangle$, $\langle r\rangle$, and $\langle m^0\rangle$. In the limit $N\to\infty$, these quantities depend solely on m_0 and α.

3.2 Overlap Distributions

As discussed in Methods, the empirical distribution $p(m)$ is bimodal and takes the general form

$$p(m) = (1-p_1)p_{m<1}(m) + p_1\delta(m-1) \tag{63}$$

where p_1 is the probability that a structure is perfectly retrieved from memory and $p_{m<1}(m)$ corresponds to the distribution of m for imperfectly retrieved memories. The shape of the distribution is characterized by p_1, the width of the lower m mode, σ_m and the mean of that mode, m^*. These quantities all depend on the initial overlap m_0 used to retrieve the memory Results for p_1, m^* and σ_m are shown in Fig. 2a, for several values of N and two values of α.

We also compare the overlap distributions for structures of length L with retrieval cue of length $L_0 = 2$ with the overlap distribution for a Pseudo-inverse network with random patterns for the corresponding values of m_0 given in Eqn. 35 in Fig. 3.

3.3 Retrieval Dynamics

When retrieving structures from memory (in the absence of noise), the update equation for the state of each neuron at time t is given by

$$\sigma_i(t) = \mathrm{sgn}\left(\sum_j J_{ij}\sigma_j(t-1)\right) \tag{64}$$

For all of the simulations in the main text, we consider parallel updates where all of the neurons $1\leq i\leq N$. We find that serial updates give qualitatively similar results to parallel updates. In general, for large N we find

$$\langle m\rangle \sim f(\alpha)m_0 \tag{65}$$

where $\langle m\rangle \to m^*$ as $N\to\infty$. We find that serial updates obey the form in Eqn. 65 with a slightly smaller value of the coefficient $f(\alpha)$. Additionally, for serial dynamics, we can consider the robustness of our results under addition of noise in the updates. To do this, we use the Metropolis algorithm to obtain the final equilibrium state, where the amplitude of the noise is controlled by the inverse temperature β. At each update, the acceptance probability is given by

$$p(\sigma_i \to -\sigma_i) = \min\left(1, e^{-2\beta h_i\sigma_i}\right) \tag{66}$$

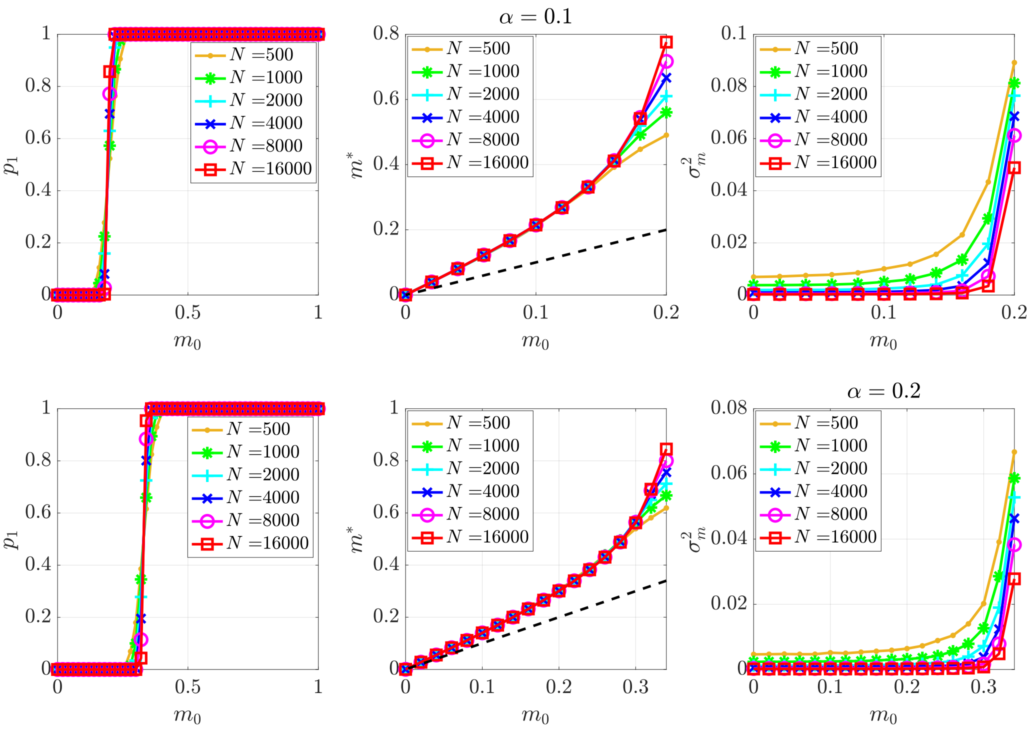

Figure 2. p_1, m^*, and σ_m^2 are shown as functions of the initial overlap m_0 for several values of N for Pseudo-inverse networks storing random memories for two values of the memory load α. The dotted black line in the middle figures show that $m^* > m_0$ for these memory loads even when the initial state is far outside of the basin of attraction. $T = 20$ parallel updates are used for memory retrieval and averages are performed over 50 trials.

We see that Eqn. 66 becomes equivalent to Eqn. 64 for the noiseless case $\beta = \infty$. In Fig. 4, we show the average overlap $\langle m \rangle$ of the network state with the cued memory as a function of time for several different initial overlaps m_0 retrieved through parallel dynamics and serial dynamics for several different values of β. In Fig. 5 we show $\langle m \rangle$, $\langle r \rangle$, $\langle m^0 \rangle$ as function of retrieval cue overlap m_0. We see that the highest overlap is achieved for parallel updates when the serial updates are done in random order (alternative orders are discussed in[2]). However, the results are qualitatively similar for the different dynamics shown, demonstrating that small amounts of noise have little effect.

In Fig. 5a and c, we see that outside of the basin of attraction of the cued memory, i.e., values of m_0 where $\langle m \rangle < 1$, the final network state retains some memory of the initial state reflected by $\langle m^0 \rangle > m_0$.

3.4 Comparing Learning Rules

In the main text, we considered storing structured memories in recurrent neural networks where the synaptic weights are set via the Pseudo-inverse rule. This learning rule fully de-correlates linearly independent memories so that each memory is a perfect fixed point for $\alpha < 1$. However, the Pseudo-inverse rule is both non-local and non-incremental. This makes it unlikely to be biologically implemented in a straightforward manner.

We now consider two learning rules that are both local and incremental. The first is the Hebb rule and the second is the Storkey rule proposed in[?,3,4] (see Eqns. (26)–(28) in Methods). The Storkey rule can be viewed as a biologically plausible approximation to the Pseudo-inverse rule for small α[4]. As a result, the storage capacity of this rule $\alpha_c \approx 0.4$ is significantly higher than the Hebb rule ($\alpha_c \approx 0.14$) and the basins of attraction are larger and more even across different memories[?].

The storage and retrieval of random memories in Hopfield networks near saturation, i.e., when the number of memories P scaling linearly in N, is limited by interference between different memories[5]. The overlaps between different memories are characterized by the following matrix

$$C^{\mu\nu} = \frac{1}{N} \sum_{i=1}^{N} \sigma_i^{\mu} \sigma_i^{\nu} \tag{67}$$

Note that $C^{\mu\nu}$ is a $P \times P$ matrix that has the form of a sample covariance matrix.

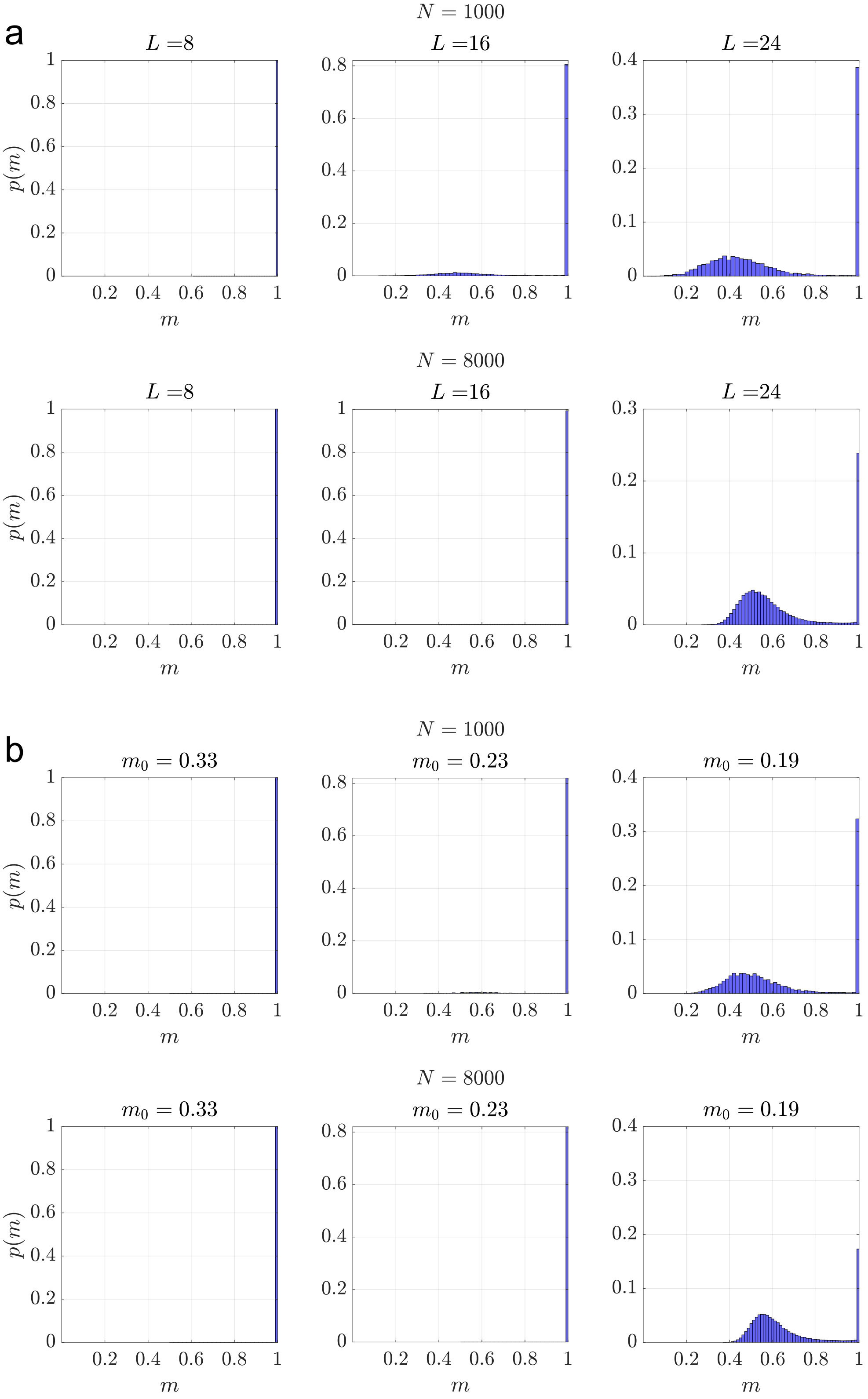

Figure 3. (a) Histograms of the final overlaps m for structures of various lengths of L for $\alpha = 0.1$ and $L_0 = 2$ and 100 trials. $T = 20$ parallel updates are used to retrieve memories. The top panel shows $N = 1000$ and bottom shows $N = 8000$. (b) Histograms for values of m_0 obtained from Eqn. 37 corresponding to $L_0 = 2$ and $L = 8, 16, 24$ (left to right).

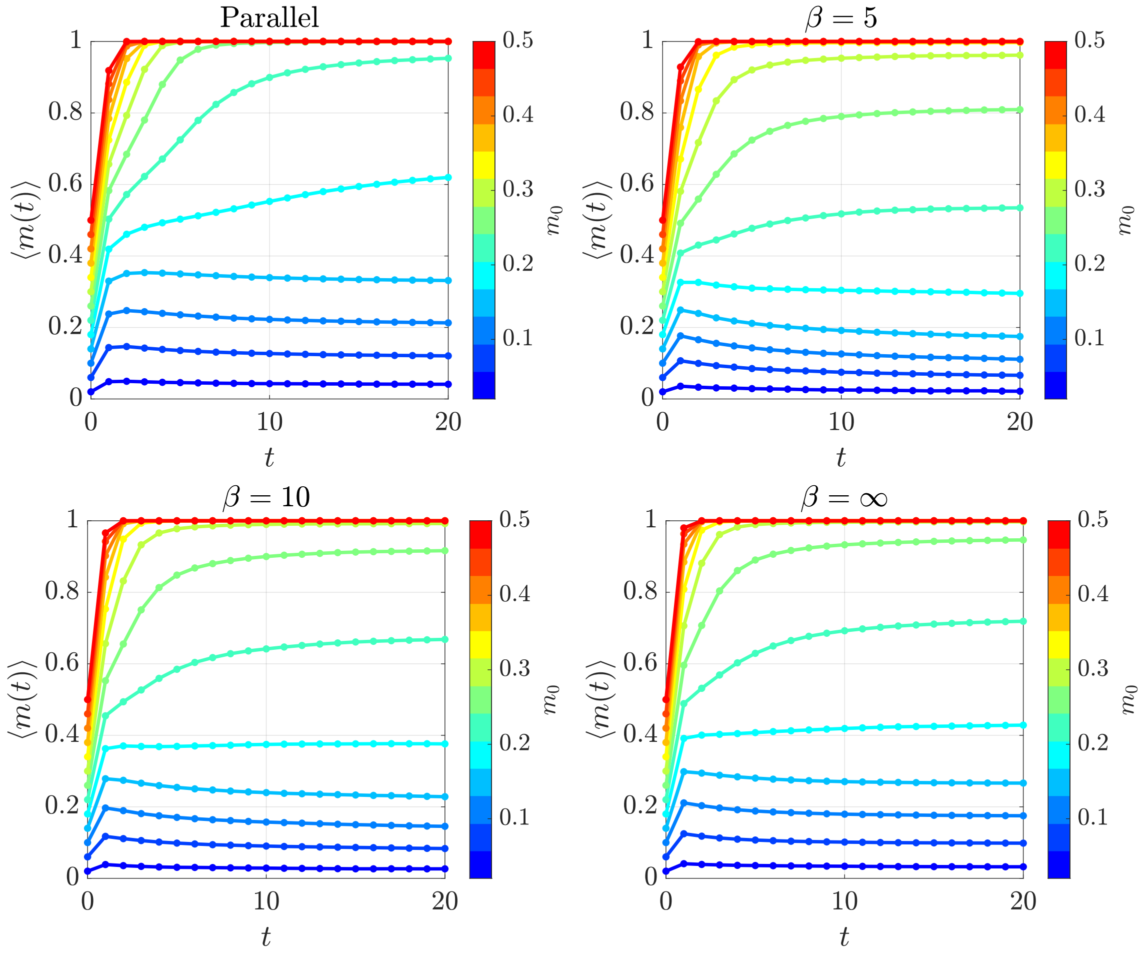

Figure 4. Evolution of the averaged overlap order parameter $\langle m \rangle$ over $T = 20$ updates compared for parallel updates serial dynamics at zero noise ($\beta = \infty$), and serial dynamics with noise amplitude determined by inverse temperature $\beta = 5, 10$, for several different initial conditions m_0. The Pseudo-inverse rule is used to store the memories. $\alpha = 0.1$, $N = 1000$, and 50 trials are used for all figures.

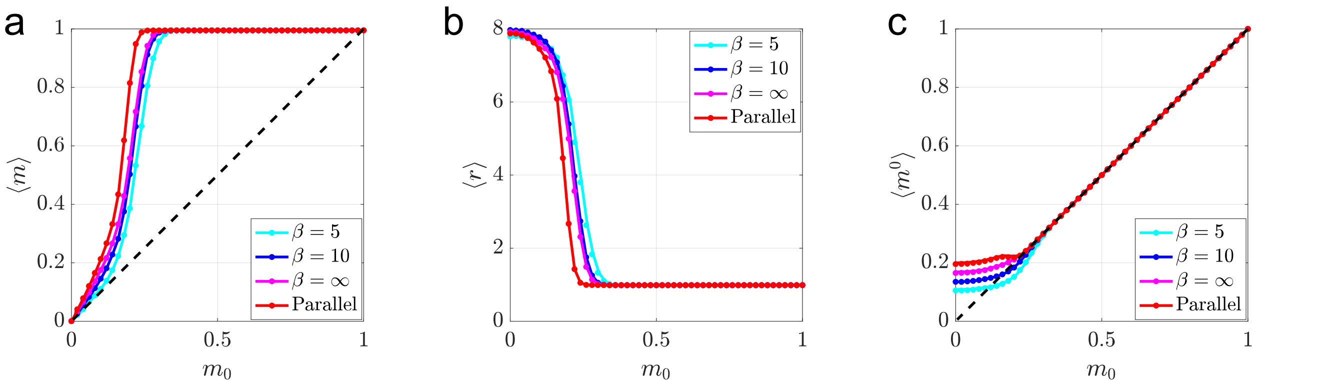

Figure 5. Comparison of averaged order parameters $\langle m \rangle$ (a), $\langle r \rangle$ (b), and $\langle m^0 \rangle$ (C), after $T = 20$ updates as functions of retrieval cue overlap m_0 are compared for parallel updates and serial updates for various values of β. The dashed line in A and C is shown to compare the value of the final overlap with the cued memory $\langle m \rangle$ and the initial state $\langle m^0 \rangle$ to the initial overlap with the cued memory. The Pseudo-inverse rule is used to store memories and $\alpha = 0.1$, $N = 1000$, 50 trials are used for all figures.

We can compare the Pseudo-inverse, Hebb, and Storkey rules by looking at the forms of the local fields given by

$$h_i(t) = \sum_j J_{ij}\sigma_j(t) \tag{68}$$

From Eqn. 64, we see that a memory is a fixed point if $\sigma_i^\mu h_i > 0$ for all $i = 1,\ldots,N$. The elimination of the self-coupling term J_{ii} greatly increases the basins of attraction of the pattern. In the limit $N,P \to \infty$ this can be accomplished by replacing J_{ij} with $J_{ij} - \alpha\delta_{ij}$[2]. The interference between memories is contained in the noise term in the local field.

We start by considering the Hebb rule, with synaptic weight matrix given by

$$J_{ij}^H = \sum_\mu^P \sigma_i^\mu \sigma_j^\mu,\ i \neq j \tag{69}$$

The local field for the Hebb rule can be expressed as

$$h_i^H(t) = \sum_{\mu=1}^P \sigma_i^\mu m^\mu(t) - \alpha\sigma_i(t) \tag{70}$$

Next we consider the the Pseudo-inverse rule with synaptic weight matrix expressed in terms of $C^{\mu\nu}$ as

$$J_{ij}^P = \sum_{\mu,\nu=1}^P \sigma_i^\mu (C^{-1})^{\mu\nu}\sigma_j^\nu,\ i \neq j \tag{71}$$

It is useful to decompose the state of the network $\sigma_i(t)$ in two parts as follows

$$\sigma_i(t) = \sum_{\mu=1}^P a^\mu(t)\sigma_i^\mu + \delta\sigma_i(t) \tag{72}$$

where $\delta\sigma_i(t)$ is orthogonal to all of the patterns ($\sum_i \sigma_i^\mu \delta\sigma_i(t) = 0$) and a^μ is related to the order parameter $m^\mu(t)$ via

$$a^\mu(t) = \sum_{\nu=1}^P (C^{-1})^{\mu\nu} m^\nu(t) \tag{73}$$

The local field for the Pseudo-inverse rule can then be expressed as

$$\begin{aligned} h_i^P(t) &= (1-\alpha)\sum_{\mu=1}^P a^\mu(t)\sigma_i^\mu - \alpha\delta\sigma_i(t) \\ &= (1-\alpha)\sum_{\mu,\nu=1}^P \sigma_i^\mu (C^{-1})^{\mu\nu} m^\nu(t) - \alpha\delta\sigma_i(t) \end{aligned} \tag{74}$$

We can see how Eqn. 74 suppresses the effects of overlaps by considering the state $\sigma_i = \sigma_i^1$. Then $a^\mu = \delta_{\mu 1}$ regardless of any correlations of the memories and $h_i^P = (1-\alpha)\sigma_i^\mu$. This implies that each memory is an eigenvector of J_{ij}^P with eigenvalue $(1-\alpha)$ so that all memories are perfect fixed points for $\alpha \geq 1$. By contrast, we see from Eqn. 70 that h_i^H becomes

$$h_i^H = (1-\alpha)\sigma^1 + \sum_{\mu=2}^P \sigma_i^1 m^\mu \tag{75}$$

contains additional noise terms of $O\left(\frac{1}{\sqrt{N}}\right)$ because of overlaps with the other patterns. This noise reduces the capacity of the network as well as the size of the basins of attraction for each memory which are both further reduced if the patterns are not random and contain correlations.

A simplified form of the Storkey rule, discussed in[?,4,6], is given by

$$\begin{aligned} J_{ij}^{S,0} &= 0 \\ J_{ij}^{S,\mu} &= J_{ij}^{\mu-1} + \frac{1}{N}\sigma_i^\mu\sigma_j^\mu - \frac{1}{N}\sigma_i^\mu h_j^\mu - \frac{1}{N}h_i^\mu\sigma_j^\mu \end{aligned} \tag{76}$$

where

$$h_i^\mu = \sum_{j=1}^{N} J_{ij}^{\mu-1} \sigma_j^\mu \tag{77}$$

Following the analysis in[6] We can find a more compact approximation for $J_{ij}^{S,P}$. We start with $P=1$. Then $J_{ij}^{S,1}$ is given by

$$J_{ij}^{S,1} = \frac{1}{N} \sigma_i^\mu \sigma_j^\mu \tag{78}$$

For $P=2$ we have

$$J_{ij}^{S,2} = \sum_{\mu=1}^{2} \frac{1}{N} \sigma_i^\mu \sigma_j^\mu - \frac{1}{N^2} \sigma_i^2 \sum_{k=1}^{N} \sigma_j^1 \sigma_k^1 \sigma_k^2 - \frac{1}{N^2} \sum_{k=1}^{N} \sigma_i^1 \sigma_k^1 \sigma_k^2 \sigma_j^2 \tag{79}$$

For P memories, keeping terms up to $O\left(\frac{1}{N^2}\right)$, J_{ij}^S can be expressed as

$$\begin{aligned} J_{ij}^S &\approx \frac{1}{N} \sum_{\mu=1}^{P} \sigma_i^\mu \sigma_j^\mu - \frac{1}{N^2} \sum_{\mu,\nu=1}^{P} \sum_{k=1}^{N} (1-\delta_{\mu\nu}) \sigma_i^\mu \sigma_k^\mu \sigma_k^\nu \sigma_j^\nu \\ &\approx \frac{1}{N} \sum_{\mu=1}^{P} (1 + \sum_{k=1}^{N} (\sigma_k^\mu)^2) \sigma_i^\mu \sigma_j^\mu - \frac{1}{N} \sum_{\mu,\nu=1}^{P} \sigma_i^\mu \left(\frac{1}{N} \sum_{k=1}^{N} \sigma_k^\mu \sigma_k^\nu \right) \sigma_j^\nu \\ &\approx \frac{2}{N} \sum_{\mu=1}^{P} \sigma_i^\mu \sigma_j^\mu - \frac{1}{N} \sum_{\mu,\nu=1}^{P} \sigma_i^\mu C^{\mu\nu} \sigma_j^\nu \end{aligned} \tag{80}$$

We can relate J_{ij}^S to the Pseudo-inverse rule given in Eqn. 71 by rewriting J_{ij}^P using the following series expansion of C^{-1}

$$\begin{aligned} C^{-1} &= (\mathbb{1} - (\mathbb{1} - C))^{-1} \\ &\approx 2\mathbb{1} - C + O(C^2) \end{aligned} \tag{81}$$

Plugging this expression back into Eqn. 71 gives us

$$J_{ij}^P \approx \frac{1}{N} \sum_{\mu=1}^{P} \sigma_i^\mu (2\delta^{\mu\nu} - C^{\mu\nu}) \sigma_j^\nu \tag{82}$$

which is identical to the expression for J_{ij}^S given in the last line of Eqn. 80 up to terms of $O\left(\frac{1}{N^2}\right)$.

The expansion of C in Eqn. 81 converges if the eigenvalues of C are all contained in the interval $[0,2]$. For random memories with components σ_i^μ drawn iid from $\mathcal{N}(0,1)$, the distribution of the eigenvalues of $C^{\mu\nu}$ is given by the Marchenko-Pastur distribution given by, which talks the following form for $P, N \to \infty$

$$\rho(\lambda) = \frac{2}{\pi} \frac{\sqrt{(\lambda_+ - \lambda)(\lambda - \lambda_-)}}{\lambda} + \mathbb{1}\{\alpha < 1\}(1-\alpha)\delta(\lambda),\ \lambda_\pm = (\sqrt{\alpha} \pm 1)^2 \tag{83}$$

Since $\lambda \in [\lambda_-, \lambda_+]$, the expansion of C is valid for $\alpha < 0.17$ implying that $J_{ij}^S \approx J_{ij}^P$ for sufficiently small α and large N.

In Fig. 6 we compare $\langle m \rangle$ as a function of m_0 for two values of α for the three learning rules and in Fig. 7, we show m_{min} (as defined in the main text) as a function of α.

3.5 Connection to Storage of Knowledge Structures

While the various learning rules and dynamics have quantitative effects on the values on the various network order parameters at fixed memory load and network size, their qualitative behavior as a function of the retrieval cue overlap m_0 remains roughly the same for small values of α below capacity.

In general, we have found that outside of the basin of attraction, $\langle m \rangle$ (and m^*) scales linearly with m_0 for all three learning rules and takes the form in Eqn. 7 where the coefficient $f(\alpha)$ depends on the learning rule.

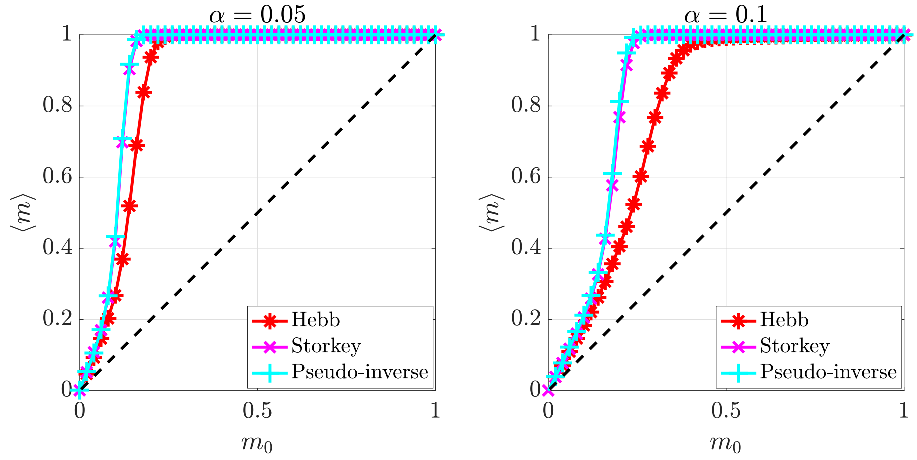

Figure 6. Comparison of $\langle m \rangle$ as function of retrieval cue overlap m_0 for the Hebb, Storkey, and Pseudo-inverse learning rules for two values of α. $T = 20$ parallel updates, $N = 1000$ and 50 trials are used for both figures.

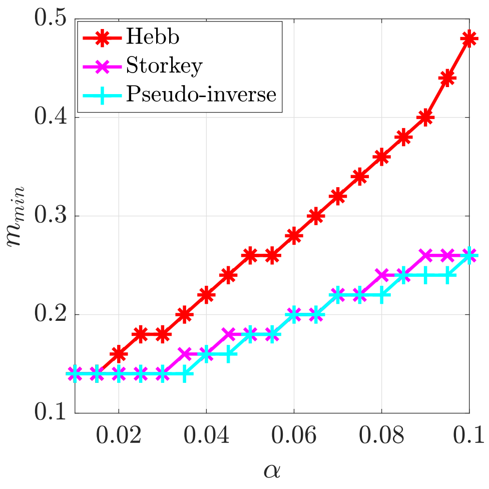

Figure 7. The average size of the basins of attraction (as defined in the main text) for the Hebb Storkey and Pseudo-inverse learning rules are shown together for small values of α.

In all cases, for large N where $\langle m \rangle \to m^*$, the SNR behaves as

$$\begin{aligned} \text{SNR} &\sim \frac{2c}{\pi}\frac{(m^*)^2}{L} \\ &\sim \frac{8cf(\alpha)^2}{\pi^3}\frac{NL_0}{L^2} \end{aligned} \tag{84}$$

As a result, while the choice of learning rule and retrieval dynamics modify the value of $f(\alpha)$ defined in Eqn. 65, they do not change the scaling behavior in Eqn. 85.

Comparing P_ε for structures stored using the Hebb, Storkey, and Pseudo-inverse rules in Fig. 8a. In general, we find that for low values of $\alpha \lesssim 0.3$, the error of decoding from memories stored via the Storkey rule is very similar to the Pseudo-inverse rule. Both are significantly lower the error obtained when decoding from memories stored using the Hebb rule. This suggests that the Storkey learning rule sufficiently decorrelates the different structures to allow for both efficient storage and retrieval of structured knowledge.

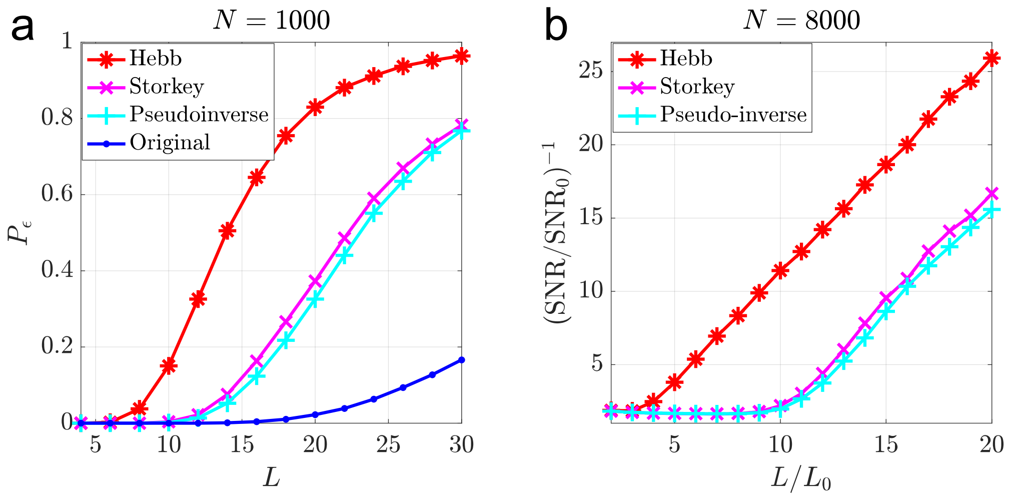

Figure 8. (a) Comparison of the decoding error P_ε for structures of size $N = 1000$ stored using the Hebb rule, Storkey rule, and Pseudo-inverse rule at memory load $\alpha = 0.1$ and retrieved from memory with a partial cue of length $L_0 = 2$. The P_ε for the original structure is also shown for comparison and the size of the dictionary is fixed to $D = 30N$. (b) $(\text{SNR/SNR}_0)^{-1}$v. L/L_0 shown for $N = 8000$. $T = 20$ parallel updates are used for both figures and the is average is performed over $20,000$ memories.

4 Temporal Sequences as Sequences of Attractors

Previously in[7,8], it was shown that a temporal sequence of memories could be stored in a Hopfield network by adding a second asymmetric synaptic interaction of the form

$$J_{ij}^{(2)} = \frac{\lambda}{N}\sum_{\ell=1}^{L}\sigma_i^{\ell+1}\sigma_j^{\ell},\ i \neq j \tag{85}$$

to the synaptic weight matrix, where $L < P$ and $\lambda > 1$.

We can store Q sequences in the network by summing together a contribution $J_{ij}^{(2,q)}$ for each sequence, i.e.,

$$J_{ij}^{(2)} = \sum_{q=1}^{Q} J_{ij}^{(2,q)} \tag{86}$$

as long as each memory is only contained in one sequence.

To retrieve a sequence, one starts with an initial state $\sigma_0 = \sigma(0)$ within the basin of attraction of the first memory in the sequence. The evolution of the network state is then given by

$$\sigma_i(t) = \text{sgn}\left(\sum_j J_{ij}^{(1)}\sigma_j(t-1) + \sum_j J_{ij}^{(2)}\bar{\sigma}_j(t-1)\right) \tag{87}$$

where $\bar{\sigma}_i(t)$ is defined as

$$\bar{\sigma}_i(t) = \int_{-\infty}^{t} dt' w(t-t')\sigma_i(t) \tag{88}$$

where the function $w(t)$ represents a dynamic memory characterized by time constant τ. A simple choice for $w(t)$ uses the heaviside function, i.e. $w(t) = \frac{1}{\tau}\Theta(t-\tau)$, which we use for the simulation results shown in Fig. 6d of the main text. $J_{ij}^{(1)}$ is given by a learning rule for the conventional Hopfield network such as the Hebb, Storkey, or Pseudo-inverse rules.

For long sequences, transitions between attractors will be equally spaced with period t_0 after initial transients and the system will be in the pattern μ in the time interval $((\mu-1)t_0, \mu t_0)$. For a heaviside function t_0 is given by

$$t_0 = \frac{\tau}{2}\left(1+\lambda^{-1}\right) \tag{89}$$

We can access the performance of this sequence memory by looking at $m^{\mu}(t)$ given in Eqn. 60 for each pattern in a sequence. Good performance requires $m^{\mu}(t) \approx 1$ during the interval $t \in ((\mu-1)t_0, \mu t_0)$ and $m^{\mu}(t) \ll 1$ for all other t.

Note that if a we store two sequences sharing items in the same memory network, transitions between attractors in the dynamics given in Eqn. 87 are no longer unique.

References

1. Plate, T. A. Distributed representations and nested compositional structure. (1994).

2. Kanter, I. & Sompolinsky, H. Associative recall of memory without errors. *Phys. Rev. A* **35**, 380–392 (1987).

3. Storkey, A. Increasing the capacity of a hopfield network without sacrificing functionality. In *Proceedings of the 7th International Conference on Artificial Neural Networks*, ICANN '97, 451–456 (Springer-Verlag, Berlin, Heidelberg, 1997).

4. Storkey, A. & Valabregue, R. A hopfield learning rule with high capacity storage of time-correlated patterns (1997).

5. Amit, D. J., Gutfreund, H. & Sompolinsky, H. Storing infinite numbers of patterns in a spin-glass model of neural networks. *Phys. Rev. Lett.* **55**, 1530–1533 (1985).

6. Storkey, A. J. Efficient Covariance Matrix Methods for Bayesian Gaussian Processes and Hopfield Neural Networks. 138 (1999).

7. Sompolinsky, H. & Kanter, I. Temporal association in asymmetric neural networks. *Phys. Rev. Lett.* **57**, 2861–2864 (1986).

8. Kleinfeld, D. & Sompolinsky, H. Associative neural network model for the generation of temporal patterns. Theory and application to central pattern generators. *Biophys. J.* **54**, 1039–1051 (1988).
